# Case-control analysis of truncating mutations in DNA damage response genes connects *TEX15* and FANCD2 with hereditary breast cancer susceptibility

**DOI:** 10.1038/s41598-017-00766-9

**Published:** 2017-04-06

**Authors:** Tuomo Mantere, Anna Tervasmäki, Anna Nurmi, Katrin Rapakko, Saila Kauppila, Jiangbo Tang, Johanna Schleutker, Anne Kallioniemi, Jaana M. Hartikainen, Arto Mannermaa, Pentti Nieminen, Riitta Hanhisalo, Sini Lehto, Maija Suvanto, Mervi Grip, Arja Jukkola-Vuorinen, Maria Tengström, Päivi Auvinen, Anders Kvist, Åke Borg, Carl Blomqvist, Kristiina Aittomäki, Roger A. Greenberg, Robert Winqvist, Heli Nevanlinna, Katri Pylkäs

**Affiliations:** 1 Laboratory of Cancer Genetics and Tumor Biology, Cancer and Translational Medicine Research Unit and Biocenter Oulu, Northern Finland Laboratory Centre Nordlab Oulu, University of Oulu, Oulu, Finland; 2 Department of Obstetrics and Gynecology, University of Helsinki and Helsinki University Hospital, Helsinki, Finland; 3 Laboratory of Genetics, Northern Finland Laboratory Centre NordLab Oulu, Oulu, Finland; 4 Cancer Genetic Unit, Service and Central Laboratory of Haematology, CHUV, Lausanne University Hospital, Lausanne, Switzerland; 5 Department of Pathology, Oulu University Hospital and University of Oulu, Oulu, Finland; 6 Departments of Cancer Biology and Pathology, Abramson Family Cancer Research Institute, Basser Research Center for BRCA, Perelman School of Medicine, University of Pennsylvania, Philadelphia, PA USA; 7 Medical Biochemistry and Genetics Institute of Biomedicine, University of Turku, Turku, Finland; 8 Microbiology and Genetics, Department of Medical Genetics, Turku University Hospital, Turku, Finland; 9 BioMediTech and FimLab Laboratories, University of Tampere, Tampere, Finland; 10 School of Medicine, Institute of Clinical Medicine, Pathology and Forensic Medicine, University of Eastern Finland, Kuopio, Finland; 11 Cancer Center of Eastern Finland, University of Eastern Finland, Kuopio, Finland; 12 Imaging Center, Department of Clinical Pathology, Kuopio University Hospital, Kuopio, Finland; 13 Medical Informatics and Statistics Research Group, University of Oulu, Oulu, Finland; 14 Department of Surgery, Oulu University Hospital and University of Oulu, Oulu, Finland; 15 Department of Oncology, Oulu University Hospital and University of Oulu, Oulu, Finland; 16 Cancer Center, Kuopio University Hospital, Kuopio, Finland; 17 Department of Oncology and Pathology, Department of Clinical Sciences Lund, Lund University, Medicon Village, Lund, Sweden; 18 Department of Oncology, Helsinki University Hospital, Helsinki, Finland; 19 Department of Oncology, University of Örebro, Örebro, Sweden; 20 Department of Clinical Genetics, University of Helsinki and Helsinki University Hospital, Helsinki, Finland

## Abstract

Several known breast cancer susceptibility genes encode proteins involved in DNA damage response (DDR) and are characterized by rare loss-of-function mutations. However, these explain less than half of the familial cases. To identify novel susceptibility factors, 39 rare truncating mutations, identified in 189 Northern Finnish hereditary breast cancer patients in parallel sequencing of 796 DDR genes, were studied for disease association. Mutation screening was performed for Northern Finnish breast cancer cases (n = 578–1565) and controls (n = 337–1228). Mutations showing potential cancer association were analyzed in additional Finnish cohorts. c.7253dupT in *TEX15*, encoding a DDR factor important in meiosis, associated with hereditary breast cancer (*p = *0.018) and likely represents a Northern Finnish founder mutation. A deleterious c.2715 + 1G > A mutation in the Fanconi anemia gene, *FANCD2*, was over two times more common in the combined Finnish hereditary cohort compared to controls. A deletion (c.640_644del5) in *RNF168*, causative for recessive RIDDLE syndrome, had high prevalence in majority of the analyzed cohorts, but did not associate with breast cancer. In conclusion, truncating variants in *TEX15* and *FANCD2* are potential breast cancer risk factors, warranting further investigations in other populations. Furthermore, high frequency of *RNF168* c.640_644del5 indicates the need for its testing in Finnish patients with RIDDLE syndrome symptoms.

## Introduction

Inability to respond properly to DNA damage leads to genetic instability, which is one of the main forces driving the onset and progression of tumorigenesis^1^. Accordingly, a significant fraction of inherited cancer predisposition syndromes results from germline mutations in DNA repair and cell cycle checkpoint control genes^2^. Particularly in breast cancer, the majority of the known susceptibility genes encode proteins with integral roles in DNA damage response (DDR), including the two major ones *BRCA1* and *BRCA2*, and a number of others, such as *PALB2*, *ATM* and *CHEK2* ^3–5^. Even in the era of whole exome sequencing, most of the genes with proposed role in breast cancer predisposition have been identified among those functioning in DNA repair related pathways, including *RECQL*, *FANCM* and *ERCC3* ^6–9^. All these genes are characterized by several, rare loss-of-function mutations acting as moderate to high-risk predisposing alleles for breast cancer, thus providing support for the “common disease-rare variant” hypothesis. However, a large proportion of the genetic factors predisposing to breast cancer still remains unknown, as currently known moderate to high-risk genes explain less than half of the familial and about 5% of the total breast cancer incidence^10, 11^.

In order to identify novel breast cancer predisposing alleles, we performed a case-control association analysis for 39 rare protein-truncating variants previously identified by targeted next-generation sequencing (NGS) of DDR genes in 189 Northern Finnish breast cancer patients with indications of hereditary disease susceptibility^12^. The use of founder populations, such as the Finns, provides an advantage for performing rare variant studies as these variants might be enriched in the population, thus representing a significant part of all predisposing mutations in the genes under investigation. This has proven to be the case for example for the *PALB2* ^3^ and *RAD50* ^13^ genes. Based on the current results, we propose a role in breast cancer predisposition for *TEX15* and *FANCD2*. Of these, *FANCD2* has a central role in the Fanconi anemia (FA) DNA repair pathway, which has previously been strongly linked to breast cancer predisposition^14^, whereas *TEX15* represents a novel susceptibility gene among the DDR factors. Functions of the encoded protein (Testis expressed 15) are still largely unknown, although it has been shown to be involved in DNA double-strand break repair during meiosis^15^. Current results also reveal several mutations in genes known to be relevant for other inherited syndromes linked to DNA repair deficiencies, thus providing integral information for the field of clinical genetics. These alterations include a founder mutation in *RNF168*: the encoded protein has previously been linked to BRCA1 recruitment to the DNA damage foci and identified as the causative gene for the recessively inherited RIDDLE syndrome^16^.

## Results

### Case-control analysis for the identification of breast cancer associated alleles

**Figure 1:**
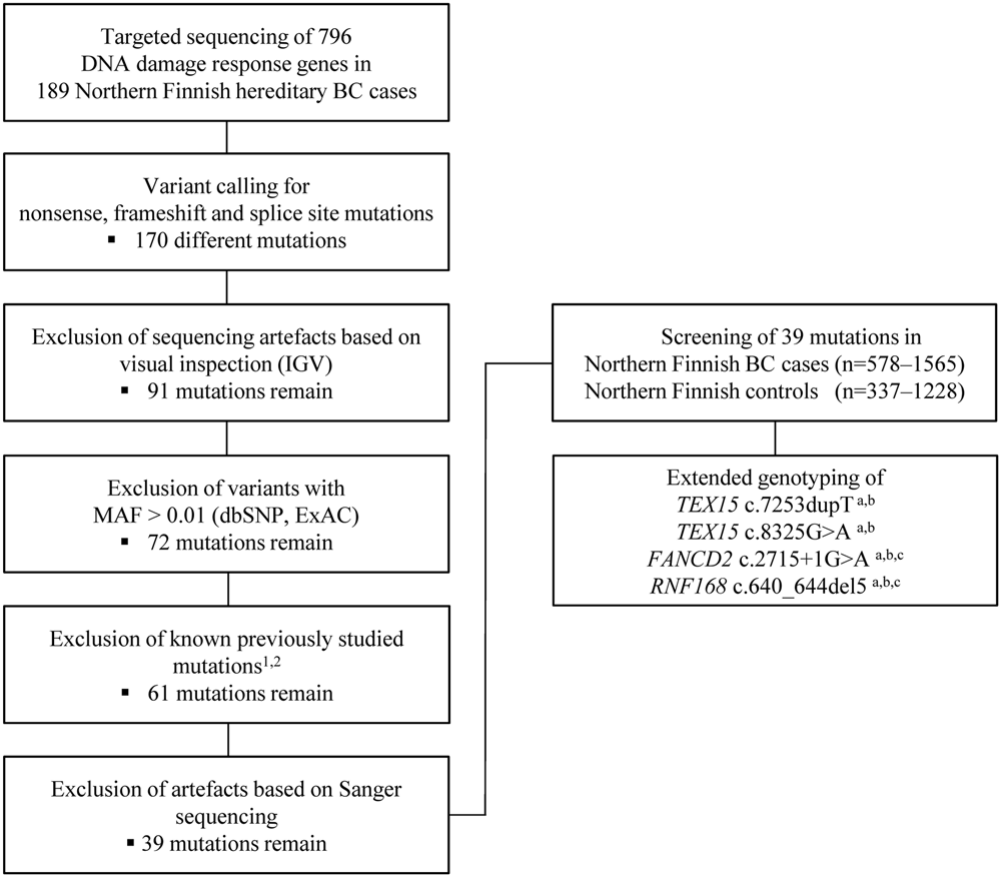
Workflow of the study. Targeted sequencing of 796 DDR genes was performed in 189 Northern Finnish breast cancer patients with indication of hereditary disease susceptibility. Analysis focused on rare mutations likely resulting in truncated protein products. After filtering steps, 39 mutations were genotyped in the Northern Finnish case and control cohorts. Extended genotyping of four mutations were performed in Finnish breast cancer and control cohorts from Helsinki^a^, Tampere^b^ and Kuopio^c^ regions. ^1^Supplementary Table S2, ^2^ *MCPH1* c.904_916del reported in Mantere *et al*.^12^. ExAC: Exome Aggregation Consortium database, BC: breast cancer, IGV: Integrative Genomics Viewer, MAF: minor allele frequency.

Filtering and validation steps presented in Fig. 1 were taken in order to select potentially disease associated protein truncating or splice site mutations for the case-control association analysis. In total, 39 different alterations met the filtering criteria (21 nonsense, 9 frameshift and 9 canonical splice site mutations) and were studied further by the case-control approach in the Northern Finnish cohorts (Supplementary Table S1). Mutations in ten genes (*CHD1L, CYP19A1, DCLRE1A, ERCC2, EXO1, GNL3, IGHMBP2, NAT10, PTPRH* and *TOP3A*) turned out to be singletons and mutations in *MSH3*, *NINL* and *ZRANB3* were present only in two patients while absent from controls, thus lacking the power for statistical comparisons. The segregation of these mutations in the carrier families could not be studied due to the lack of additional DNA samples from suitable family members, leaving their impact on breast cancer risk unknown. In total, four of the recurrent mutations showed significant (*TEX15* c.7253dupT and *FANCD2* c.2715 + 1G > A) or borderline association (*TEX15* c.8325G > A and *RNF168* c.640_644del5) with hereditary breast cancer in the Northern Finnish cohort. These mutations were further genotyped in expanded set of Finnish breast cancer cases and controls originating from Helsinki, Kuopio and Tampere (Table 1, and meta-analyses in Table 2). Of note, two of the studied mutations were also simultaneously identified in Helsinki: *FANCD2* c.2715 + 1G > A in clinical panel sequencing of a patient with early onset triple-negative breast cancer and *RNF168* c.640_644del5 in exome sequencing of hereditary breast cancer patients.

**Table 1:** Frequency of *TEX15*, *FANCD2* and *RNF168* mutations in the studied Finnish case-control cohorts.

Mutation	Study cohort	Cohort	N	WT	(%)	Mut	(%)	OR	95% CI	*p* ^a^
*TEX15*	Oulu	Hereditary BC	247	244	(98.8)	3	(1.2)	14.6	1.5–141.1	0.018
c.7253dupT		*BRCA1/2* neg.	228	225	(98.7)	3	(1.3)	15.9	1.6–153.1	0.014
Leu2418PhefsTer6		Unselected BC	1317	1314	(99.8)	3	(0.2)	2.7	0.3–26.1	0.627
rs760604179		All BC	1564	1558	(99.6)	6	(0.4)	4.6	0.6–38.1	0.149
		Controls	1190	1189	(99.9)	1	(0.1)			
	Tampere	Hereditary BC	87	87	(100)	0	(ND)	NA	NA	NA
		Controls	93	93	(100)	0	(ND)			
	Helsinki	Hereditary BC	581	581	(100)	0	(ND)	NA	NA	NA
		Controls	640	640	(100)	0	(ND)			
*TEX15*	Oulu	Hereditary BC	247	240	(97.2)	7^b^	(2.8)	2.7	1.1–6.8	0.063
c.8325G > A		*BRCA1/2* neg.	228	222	(97.4)	6	(2.6)	2.5	0.9–6.6	0.104
Trp2775Ter		Unselected BC	1318	1308	(99.2)	10	(0.8)	0.7	0.3–1.6	0.411
rs146619272		All BC	1565	1548	(98.9)	17^b^	(1.1)	1.0	0.5–2.1	1.000
		Controls	1203	1190	(98.9)	13	(1.1)			
	Tampere	Hereditary BC	87	86	(98.9)	1	(1.1)	NA	NA	0.481
		Controls	94	94	(100)	0	(ND)			
	Helsinki	Hereditary BC	986	977	(99.1)	9^c^	(0.9)	1.0	0.4–2.5	1.000
		Controls	1088	1078	(99.1)	10	(0.9)			
*FANCD2*	Oulu	Hereditary BC	247	244	(98.8)	3^b^	(1.2)	7.5	1.3–45.3	0.036
c.2715 + 1G > A		*BRCA1/2* neg.	228	226	(99.1)	2	(0.9)	5.4	0.8–38.7	0.118
E906LfsX4		Unselected BC	1152	1150	(99.8)	2	(0.2)	1.1	0.2–7.6	1.000
rs201811817		All BC	1399	1394	(99.6)	5^b^	(0.4)	2.2	0.4–11.3	0.459
		Controls	1228	1226	(99.8)	2	(0.2)			
	Tampere	Hereditary BC	87	87	(100)	0	(ND)	NA	NA	1.000
		Unselected BC	646	644	(99.7)	2	(0.3)	1.2	0.2–8.5	1.000
		All BC	733	731	(99.7)	2	(0.3)	1.0	0.1–7.4	1.000
		Controls	767	765	(99.7)	2	(0.3)			
	Kuopio	Unselected BC	668	668	(100)	0	(ND)	NA	NA	NA
		Controls	156	156	(100)	0	(ND)			
	Helsinki	Hereditary BC	1175	1171	(99.7)	4	(0.3)	2.2	0.4–11.9	0.436
		Unselected BC	1727	1723	(99.8)	4^d^	(0.2)	1.5	0.3–8.1	1.000
		All BC	2513	2506	(99.7)	7	(0.3)	1.8	0.4–8.6	0.727
		Controls	1272	1270	(99.8)	2	(0.2)			
*RNF168*	Oulu	Hereditary BC	247	243	(98.4)	4^b^	(1.6)	3.2	0.9–11.5	0.077
c.640_644del5		*BRCA1/2* neg.	228	225	(98.7)	3	(1.3)	2.6	0.7–10.6	0.165
Lys214Terfs		Unselected BC	1194	1185	(99.2)	9	(0.8)	1.5	0.5–4.2	0.606
rs777601326		All BC	1441	1428	(99.1)	13^b^	(0.9)	1.8	0.7–4.7	0.257
		Controls	1185	1179	(99.5)	6	(0.5)			
	Tampere	Hereditary BC	87	87	(100)	0	(ND)	NA	NA	0.576
		Unselected BC	445	444	(99.8)	1	(0.2)	0.2	0.02–1.4	0.073
		All BC	532	531	(99.8)	1	(0.2)	0.1	0.01–1.1	0.048
		Controls	413	409	(99.0)	4	(1.0)			
	Kuopio	Unselected BC	652	635	(97.4)	17	(2.6)	0.6	0.3–1.2	0.157
		Controls	288	275	(95.5)	13	(4.5)			
	Helsinki	Hereditary BC	1170	1163	(99.4)	7	(0.6)	0.7	0.3–1.8	0.486
		Controls	1272	1261	(99.1)	11	(0.9)			

^a^ *p*-value of χ^2^ test or Fisher’s exact test, ^b^one *BRCA1/2* carrier, ^c^one homozygote, ^d^416 of the hereditary patients belong also to the unselected cohort, includes one carrier. BC: breast cancer, *BRCA1/2* neg: includes only the hereditary cases negative for pathogenic *BRCA1/2* mutations, CI: confidence interval, Mut: mutation carrier, NA: not analyzed, ND: not detected, OR: odds ratio, WT: wild type.

**Table 2:** Meta-analyses combining Oulu, Tampere, Kuopio and Helsinki screening results.

Mutation^a^	Cohort^b^	OR	95% CI	*p*-value^c^	Oulu Mut/WT	Tre Mut/WT	Kuo Mut/WT	Hki Mut/WT
*TEX15*	All BC	1.0	0.6–1.8	0.959	16/1530	1/86	NA	9/977
c.8325G > A	Hereditary BC	1.6	0.8–3.1	0.208	6/222	1/86	NA	9/977
rs146619272	Controls				13/1190	0/94	NA	10/1078
*FANCD2*	All BC	1.6	0.6–4.1	0.375	4/1376	2/731	0/668	7/2506
c.2715 + 1G > A	Hereditary BC	2.6	0.8–9.1	0.131	2/226	0/87	NA	4/1171
rs201811817	Unselected BC	1.3	0.4–3.6	0.675	2/1150	2/644	0/668	4/1723
	Controls				2/1226	2/765	0/156	2/1270
*RNF168*	All BC	0.7	0.5–1.2	0.211	12/1410	1/531	17/635	7/1163
c.640_644del5	Hereditary BC	0.9	0.4–2.0	0.757	3/225	0/87	NA	7/1163
rs777601326	Unselected BC	0.7	0.4–1.3	0.241	9/1185	1/444	17/635	NA
	Controls				6/1179	4/409	13/275	11/1261

^a^ *TEX15* c.7253dupT was excluded from the meta-analysis since the mutation was present only in the Northern Finnish cohort. ^b^All the known *BRCA1/2* carriers were excluded from the meta-analyses. ^c^Logistic regression model, all the analyzed cohorts combined and all individual datasets exploited. CI: confidence interval, BC: breast cancer, Hki: Helsinki, Kuo: Kuopio, Mut: mutation carrier, NA: not available, OR: odds ratio, Tre: Tampere, WT: wild type.

### *TEX15* c.7253dupT mutation is stable at mRNA level and associates with breast cancer

Two different protein truncating mutations in the *TEX15* gene showed potential association with breast cancer in the Northern Finnish cohort. *TEX15* encodes a protein that has been suggested to have a specific role in DNA double-strand break repair, as it is necessary for formation of DMC1 and RAD51 foci on meiotic chromosomes^15^. *TEX15* has initially been classified into the group of cancer/testis (CT) antigen encoding genes activated in various cancers, and its expression has been considered to be more or less strictly restricted to testis among mature organs^17, 18^. However, besides testis, low level *TEX15* expression has been reported also in other normal tissues (including uterus, brain and smooth muscle), whereas in cancer samples *TEX15* expression has been recurrently observed in breast, lung and bladder cancer, and cutaneous melanoma^17, 19, 20^. We confirmed that *TEX15* was expressed in the tested patient-derived LCLs and also in breast epithelial cancer cells (MCF7).

The *TEX15* frameshift mutation (c.7253dupT, Leu2418PhefsTer6, rs760604179) was observed in three cases from the Northern Finnish hereditary cohort (3/247, 1.2%), whereas only one carrier was identified in controls (1/1190, 0.1%, *p = *0.018, odds ratio [OR] = 14.6 and 95% confidence interval [CI] = 1.5–141.1) (Table 1). All three cases were diagnosed with breast cancer at young age (39, 36 and 38) and did not have mutations in previously screened cancer predisposition genes^3, 21, 22^. Curiously, two of them had a father diagnosed with prostate cancer: one was confirmed as a *TEX15 *c.7253dupT carrier, the other was not available for testing (Supplementary Fig. S1a). The mother of the third index case was diagnosed with breast cancer at the age of 40 years (carrier status unknown). Three more carriers (diagnosed at the age of 37, 58 and 47, respectively) were identified in the unselected cohort (Supplementary Fig. S1a). Only one sample from family members was available for mutation testing: the mother of the index (family 6), diagnosed with bone marrow cancer at the age of 81, was confirmed as a carrier. Data from pathology reports was available for all six carriers. Tumors were mainly ER/PR positive, and in five carriers these were of ductal origin (Supplementary Table S2). No additional *TEX15* c.7253dupT carriers were identified in either the cases or the controls originating from Helsinki or Tampere (both Southern Finland), indicating that the mutation is geographically restricted to Northern Finland.

Another *TEX15* truncating mutation (c.8325G > A, Trp2775Ter, rs146619272) was identified in 7/247 (2.8%) of the Northern Finnish hereditary cases and it showed a borderline association with breast cancer (*p = *0.063, OR = 2.7 and 95% CI = 1.1–6.8). The enrichment was restricted to the hereditary cohort, whereas the prevalence of the mutation in the unselected cases was not different from the controls (Table 1). Further screening of *TEX15* c.8325G > A in the additional Helsinki and Tampere cohorts revealed several carriers in both hereditary cases and controls with similar frequencies, indicating that *TEX15* c.8325G > A might not be a breast cancer risk factor or that the risk associated with it is low (Table 1 and Table 2).

**Figure 2:**
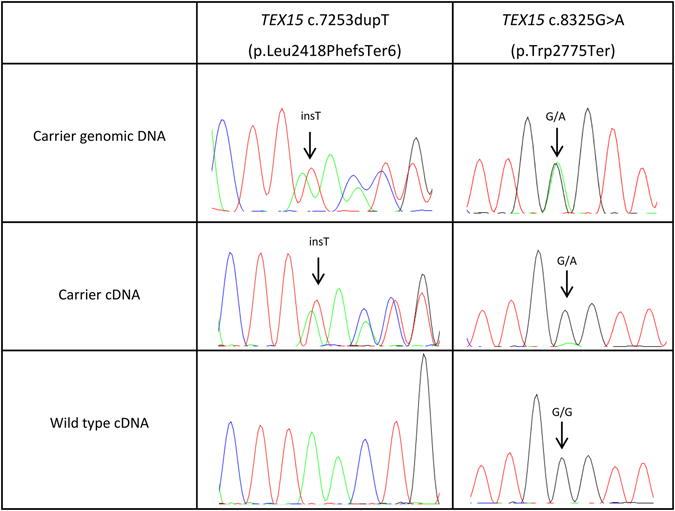
Sequencing of *TEX15* c.7253dupT and c.8325G > A mutation sites at genomic DNA and cDNA level.

To resolve this apparent discrepancy in breast cancer association between the two protein truncating mutations in the same gene, we tested their effect at mRNA level. Curiously, *TEX15 *c.7253dupT and c.8325G > A behaved differently: the breast cancer associated *TEX15 *c.7253dupT mRNA was stable, whereas the transcript from the c.8325G > A allele was efficiently targeted by nonsense-mediated decay (Fig. 2). This indicates that the effect of c. 8325G > A is at the dosage level of TEX15, whereas c.7253dupT could disturb at least some cellular functions of TEX15, potentially in dominant–negative manner. Although we were able to study the effect of the *TEX15 *c.7253dupT mutation at mRNA level, the presence of the truncated TEX15 protein product could not be confirmed due to lack of a suitable antibody.

As defects in some DDR genes have been associated with increased genomic instability^12, 13, 23^, we tested the prevalence of chromosomal aberrations in the *TEX15* c.7253dupT carriers. They exhibited an increase in the frequency of spontaneous chromosomal rearrangements when compared to controls, however, the difference showing only a borderline significance (p = 0.067) (Supplementary Table S3). All observed chromosomal aberrations were considered random, as no preference for specific break site or evidence for clonality was observed, thus suggesting a plausible effect on genomic instability for the heterozygous *TEX15* c.7253dupT mutation.

### Fanconi anemia allele in *FANCD2* shows enrichment in breast cancer cases

A splice site mutation (c.2715 + 1G > A, E906LfsX4, rs201811817) in the Fanconi anemia (FA) gene, *FANCD2*, showed enrichment in the Northern Finnish hereditary cohort (3/247, 1.2%) when compared to controls (*p = *0.036, OR = 7.5 and 95% CI = 1.3–45.3) (Table 1). This mutation induces the use of a cryptic splice-donor site downstream the exon (c.2715 + 28_29) resulting in inclusion of a 27 bp sequence of the intron into mRNA, which translates into a premature stop codon after three inserted amino acids. cDNA-specific sequencing of the mutation carrier LCL mRNA confirmed the expression and at least partial stability of the mutated allele (Supplementary Fig. S2). The deleterious effect of the mutation for the protein function is supported by its occurrence in two FA patients at compound heterozygous state^24^. All three c.2715 + 1G > A carriers from the hereditary cohort were diagnosed with breast cancer at relatively young age (39, 40 and 42). In addition to several breast cancer cases in their families, two index cases had also a close relative (1st or 2nd degree) diagnosed with blood cancer (at the age of 1 and 17 years, respectively) (Supplementary Fig. S1b). However, the segregation of the *FANCD2* mutation with the disease remained inconclusive due to the lack of additional DNA samples from the family members. Of note, one index case was a double mutant for *FANCD2* c.2715 + 1G > A and *BRCA1* c.4097-2 A > G (rs80358019), and curiously there were also several healthy carriers of both mutations in this family. Further studies on additional Finnish breast cancer cohorts (both unselected and hereditary) identified more *FANCD2* c.2715 + 1G > A carriers (Tables 1 and 2), but the frequency remained low, which is typical for mutations causative of FA^25^. In Helsinki, the *FANCD2* mutation was identified in four breast cancer cases in the genotyped hereditary cohort (diagnosed at the age of 41, 44, 49 and 68, respectively). Two of the index cases were diagnosed also with basal cell carcinoma. However, additional DNA samples were available only in one family, in which the mutation did not segregate with breast cancer (Supplementary Fig. S1c). Data from pathology reports was available for eleven carriers, the tumors being mainly ductal (9/11). Curiously, three of the tumors (27%) were triple-negative (ER-/PR-/HER2-, Supplementary Table S2), a subtype that has previously been associated with defects in other DDR genes^26^. For the unselected carriers from all of the analyzed cohorts, the mean age at diagnosis was 57 years (variation 48–66 years). In the meta-analysis, the mutation had an OR of 2.6 among the *BRCA1/2* mutation negative hereditary cases when compared to controls, but the association remained under the level of statistical significance (Table 2).

### Mutations in genes causative for other inherited syndromes

Besides the FA gene *FANCD2*, targeted sequencing of DDR genes revealed several truncating variants in genes known to result in recessively inherited syndromes with variable phenotypes, including sensitivity to UV-light, impairment of eyesight or muscle function and also susceptibility to various cancers (OMIM http://www.omim.org/): *APTX*, *CEP164*, *CYP19A1*, *ERCC2*, *ERCC6*, *IGHMBP2*, *NTHL1*, *RNF168* and *UVSSA*. The observed recurrent heterozygous alleles in these genes did not associate with breast cancer, although the association for the singleton mutations in *CYP19A1*, *ERCC2* and *IGHMBP2* could not be excluded (Supplementary Table S1). Among this group of syndrome genes, *RNF168* was particularly interesting since it encodes an E3 ubiquitin-protein ligase involved in the signaling pathway that is required to recruit BRCA1 to the site of DNA damage^16^. In addition, biallelic *RNF168* truncating mutations have previously been reported in two cases with recessively inherited RIDDLE syndrome (Radiosensitivity, Immunodeficiency, Dysmorphic features and Learning Difficulties)^16^. The currently identified *RNF168* allele (c.640_644del5, Lys214Terfs, rs777601326) was observed in 4/247 (1.6%) of the Northern Finnish hereditary cases, but it was also detected in 6/1185 (0.5%) of the controls and showed only a borderline association with breast cancer susceptibility (*p = *0.077, OR = 3.2 and 95% CI = 0.9–11.5) (Table 1). The prevalence of the *RNF168* c.640_644del5 allele was also tested in Helsinki, Tampere and Kuopio case-control cohorts. Unexpectedly, it was observed at higher frequency in controls than in breast cancer patients in all three series and showed no association with breast cancer in the meta-analysis (Table 2). Furthermore, it had a strikingly high carrier frequency in Kuopio (Eastern Finland) not only in cases (2.6%) but also in controls (4.5%) (Table 1), indicating that homozygotes for this mutation should be expected in the Finnish population.

## Discussion

The extensive case-control association analysis performed here for the rare, presumably deleterious alleles in genes encoding important players in the DDR pathway identified two potentially breast cancer predisposing mutations: *TEX15 *c.7253dupT and *FANCD2* c.2715 + 1G > A. Whereas FANCD2 has an integral role in the canonical FA pathway, the biological functions of TEX15 are only gradually emerging. However, TEX15 has already been shown to operate in homologous recombination and to guide the loading of RAD51 to the sites of DNA double-strand breaks along with BRCA1/2 during male meiosis^15, 27^. Reflecting this, biallelic mutations in *TEX15* lead to spermatogenic failure in both men and mice^15, 28^. Despite these testis-specific functions, we confirmed the *TEX15* expression in LCLs and MCF7 cell line. Together with the observed association with breast cancer this indicates that the role of TEX15 extends beyond testis and meiosis-specific double-strand break repair.

The breast cancer associated *TEX15 *c.7253dupT differed from the other observed *TEX15* mutation, c.8325G > A, in that it was stable at mRNA level whereas the c.8325G > A mRNA was efficiently degraded. It is possible that this stable allele might disturb some of the normal cellular functions of TEX15, potentially relating to cancer predisposition, more severely than a complete null allele. Both c.7253dupT and c.8325G > A mutations reside in the same long exon (altogether 1259 base pairs), preceding the last one. The degraded *TEX15* null mutation (c.8325G > A) creates a stop codon close to the original, whereas the stop codon eventually created by the c.7253dupT frameshift is more distant. According to a recent paper by Lindeboom *et al*.^29^, the distance of a new stop codon from the original one and the long length of the exon where the mutation resides are the key factors for defining the efficiency of nonsense-mediated decay. Indeed, this could provide a plausible explanation to the currently observed situation, which is actually analogous to that observed for the Finnish *PALB2* and *MCPH1* founder mutations. Both of them reside in a long exon of the gene, are stable at mRNA level, and are more common in people affected with breast cancer compared to healthy controls^12, 23^. Besides introducing *TEX15* as a putative new breast cancer predisposition gene, the current results can be of relevance for individuals suffering from idiopathic spermatogenic failure. The *TEX15* null allele (c.8325G > A) has a carrier frequency of approximately 1% in the Finnish population, and individuals homozygous for this mutation are likely to suffer from spermatogenic failure as in the family described by Okutman *et al*.^28^.

The prevalence of *FANCD2* c.2715 + 1G > A mutation among the studied cohorts supports the previously established link between FA and breast cancer: monoallelic mutations in *BRCA2/FANCD1*, *BRIP1/FANCJ, PALB2/FANCN*, *RAD51C/FANCO* and *BRCA1/FANCS* genes predispose to breast and/or ovarian cancer, while their biallelic mutations cause FA, characterized by genomic instability, bone marrow failure, developmental abnormalities and increased incidence of cancer, particularly leukemia^14, 30, 31^. Breast cancer associated genes from the FA pathway function in the homologous recombination repair part of the pathway, while mutations in the core complex FA genes have not been associated with breast cancer^25, 32^, the exceptions potentially being *FANCM* ^8, 33^ and *FANCC* ^34^. Curiously, also in the study by Thompson *et al*. 2012, one of the index cases with truncating *FANCC* mutation carried a deleterious *BRCA2* mutation^34^, analogous to the situation observed in the current study, where one *FANCD2* and *BRCA1* double mutant was identified. Although high FANCD2 expression levels in breast tumors have previously been linked to poor survival of the patients^35^, and one common *FANCD2* SNP has been associated with sporadic breast cancer risk^36^, to our knowledge, this study is the first one reporting *FANCD2* mutations in the context of familial breast cancer.

This study also provides integral information for the field of clinical genetics as many biallelic mutations in DDR genes are known to cause severe congenital disorders. Particularly interesting is the recurrent *RNF168* c.640_644del5 mutation, which clearly represents a founder mutation in Finland, but whether it is causative for RIDDLE syndrome remains to be demonstrated. Based on the observed carrier frequency, and if the mutation is not embryonically lethal when homozygous, it is likely that RIDDLE syndrome patients exist in Finland, thus warranting further investigations. Only two patients with this condition have so far been described worldwide^16, 37^ and as the phenotype of even these patients is different, it is possible that the severity of the condition might vary. For rare conditions like RIDDLE syndrome, the identification of additional patients is important for the better characterization and understanding of the disease phenotype and etiology, and it may ultimately lead to better diagnosis and potential treatment.

Overall, this study shows that many heterozygous protein-truncating mutations in DDR genes identified in breast cancer cases are also frequently found in the healthy population and thus are not high-risk susceptibility factors for breast cancer, although any low-penetrance effects cannot be excluded. Our results indicate a potential role in breast cancer predisposition for heterozygous truncating mutations in *FANCD2* and *TEX15*. Based on our results, *FANCD2* c.2715 + 1G > A might act as a moderate breast cancer risk allele, adding *FANCD2* to the list of shared genes between FA and breast cancer. According to the ExAC database^38^, *FANCD2* c.2715 + 1G > A occurs also outside Finland and should be studied further in larger sample sets to clarify its role in breast cancer predisposition. *TEX15* represents yet another potential breast cancer susceptibility gene from the DDR pathway. Even though the currently identified *TEX15 *c.7253dupT most likely represents a Northern Finnish founder mutation, other truncating mutations, and their effect at mRNA level, warrant further studies in other populations.

## Materials and Methods

### Targeted sequencing of 796 DDR genes in Northern Finnish breast cancer patients

Initial sequencing was previously performed for 189 Northern Finnish breast cancer patients with indication of hereditary disease susceptibility [62 cases with young disease onset (≤40y), and 127 cases with family history of breast or breast and ovarian cancer]^12^. Selection and sequencing of the 796 DDR genes (Supplementary Table S4), and annotation of the variants are described in Mantere *et al*.^12^. Briefly, the selected genes encoded (1) proteins identified as being part of DNA repair processes using the GeneOntology searches and STRING v.9.0 (n = 612)^39, 40^, and (2) novel BRCA1 and PALB2 interacting proteins identified in protein complex purification assays performed with epitope tagged versions of the proteins in HeLaS3 cells (n = 184). wANNOVAR^41^, SureCall (Agilent technologies, Santa Clara, CA, USA) and Integrative Genomics Viewer (IGV)^42^ were used for annotation and visualization of the variants. All variants selected for further studies were confirmed by Sanger sequencing (ABI3130xl, Applied Biosystems, Foster City, CA, USA).

### Case-control cohorts

#### Oulu cohort (Northern Finland)

The hereditary breast cancer cohort (n = 247) consisted of 166 familial and 81 young breast cancer cases (includes familial and young cases from the discovery cohort). The familial cases were affected index individuals of Northern Finnish breast, or breast and ovarian cancer families. Inclusion criteria were the following: 1) three or more breast and/or ovarian cancers in first- or second-degree relatives (n = 86); 2) two cases of breast, or breast and ovarian cancer in first- or second-degree relatives. Of these at least one had early disease onset (<35 years), bilateral breast cancer, or multiple primary tumors including breast or ovarian cancer in the same individual (n = 26); or 3) two cases of breast cancer in first- or second-degree relatives (n = 54). Young breast cancer cases were unselected for a family history of cancer and diagnosed with breast cancer at or below the age of 40 (median 38, variation 25–40 years). These cases were combined with the familial cohort to form a single hereditary cohort, based on the assumption that when a woman below the age of 40 years develops breast cancer, a hereditary predisposition may be suspected regardless of the family history^43^. In the hereditary cohort fulfilling the inclusion criteria of the current study, 19/247 (8%) of the cases carried known mutations in *BRCA1* or *BRCA2* ^21^, Supplementary Table S5. These were included in the analysis in order to identify additional risk factors, as in some families *BRCA1/2* mutations could not alone explain the family burden of the disease, and also several individuals with double mutations in integral DDR genes have previously been described in the literature^3, 12, 34^. The unselected breast cancer cohort consisted of 1326 consecutive cases operated at the Oulu University Hospital during 2000–2014. They were unselected for a family history of cancer and age at disease onset. Altogether 1228 healthy geographically matched Finnish Red Cross blood donors (758 females and 470 males) were used as population controls.

#### Helsinki cohort (Southern Finland)

The unselected breast cancer cohort from Helsinki was collected consecutively at Helsinki University Hospital Department of Oncology in 1997–1998 and 2000^44, 45^ and Department of Surgery in 2001–2004^46^. The unselected cohort consisted of 1727 patients with invasive breast cancer. Altogether 416 patients from the unselected series had a family history of breast cancer and were included also in the hereditary breast cancer cohort. Additional familial breast cancer patients were collected at Departments of Oncology and Clinical Genetics as previously described^46, 47^. In total, the hereditary breast cancer cohort consisted of 1175 patients. Of these, 612 patients were from families with three or more breast or ovarian cancers among first- or second-degree relatives and 563 patients had one affected first-degree relative. The familial breast cancer patients have been tested negative at least for *BRCA1/2* founder mutations. Geographically matched 1272 healthy Finnish Red Cross blood donors were used as controls. All patients and controls were females.

#### Tampere cohort (Southern Finland)

The hereditary cases from Tampere consisted of 87 index cases of *BRCA1/2* mutation negative hereditary breast, or breast and ovarian cancer families as described in Kuusisto *et al*.^48^. The unselected breast cancer cases (n = 646) were collected during 1997–1999 at the Tampere University Hospital^44^. 767 geographically matched female Finnish Red Cross blood donors were used as controls.

#### Kuopio cohort (Eastern Finland)

Altogether 487 cases and 288 controls were available from the Kuopio Breast Cancer Project (KBCP)^49^. The KBCP material includes prospective breast cancer cases (unselected for the family history of breast cancer) from the province of Northern Savo in Eastern Finland, diagnosed at the Kuopio University Hospital between 1990 and 1995. The control individuals in KBCP were selected from the National Population Register at the same time interval and were matched for sex, age and long-term area of residence for the cases. Additional set of unselected breast cancer cases (n = 181) were available from the ILRS breast cancer cohort. The ILRS cases were diagnosed and collected at the Kuopio University Hospital during 2011–2014. Altogether these accounted for 668 unselected breast cancer cases and 288 controls.

Genomic DNA extracted from peripheral blood was used for mutation screening from all samples. The study was carried out with informed consent from all the participating individuals. The study was approved by the Ethical Board of the Northern Ostrobothnia Health Care District, the Ethical committee of Helsinki University Central Hospital, the Ethical Committee of Tampere University Hospital and the National Authority for Medicolegal Affairs, the Ethical committee of the University of Eastern Finland and Kuopio University Hospital and the Ministry of Social Affairs and Health in Finland. The study and the methods were conducted in accordance with the approved guidelines.

### Mutation screening

Case-control comparisons for Oulu, Kuopio and Tampere cohorts were performed by High Resolution Melt (HRM) analysis (CFX96, Bio-Rad, Hercules, CA, USA) using Type-It HRM reagents (Qiagen, Hilden, Germany). A positive control was included in all analyses, and samples with differing melting curves were further confirmed by Sanger sequencing (ABI3130xl, Applied Biosystem, Foster City, CA, USA). The Helsinki cohorts were genotyped using Custom TaqMan SNP Genotyping Assays and TaqMan Genotyping MasterMix (ThermoFisher Scientific, Waltham, MA, USA). PCR was performed on 7500 Fast Real-Time PCR System or 9800 Fast Thermal Cycler and genotypes were analyzed on 7500 Fast System SDS software v1.3.1 or 7500 software v2.0.6 (Applied Biosystems, Waltham, MA, USA). Heterozygous carriers were included as positive controls in all analyses.

### Chromosomal analysis

Chromosomal analysis of six heterozygous *TEX15 *c.7253dupT carriers [three affected index cases, one male diagnosed with prostate and skin cancer, and two healthy females (aged 25 and 58)] and nine wild type controls was carried out on metaphases obtained from regular short-term 3-day cultures of peripheral blood T-lymphocytes^13^. The blood samples of the patients selected for chromosomal analysis were collected at least 5 years after the initial breast cancer diagnosis and treatment. The controls were healthy female individuals. A minimum of 50 Giemsa-banded metaphases for each sample were evaluated by light microscopy and photographed with an automatic chromosome analyzer (CytoVision version 7.2, Applied Imaging). Chromosomal aberrations were divided into five classes as previously described^13^.

### mRNA analysis

Total RNA was isolated from MCF7 breast cancer cell line and Epstein-Barr virus transformed lymphoblastoid cell lines (LCLs) of *TEX15 *c.7253dupT, *TEX15 *c.8325G > A and *FANCD2* c.2715 + 1G > A mutation carriers and wild type controls using RNeasy Mini Kit (Qiagen, Hilden, Germany) and reverse transcribed with iScript cDNA synthesis Kit (Bio-Rad, Hercules, CA, USA). The expression and outcome of different mutations at mRNA level was studied using cDNA specific sequencing in LCLs (primers in Supplementary Table S6). TEX15 expression in MCF7 cell line was tested by cDNA specific amplification.

### Statistical analysis

Carrier frequencies between cases and controls were compared using Fisher’s exact or χ^2^ test. Mann-Whitney U-test was used to determine the difference in the number of different chromosomal aberrations between *TEX15* c.7253dupT carriers and controls. The meta-analyses of the mutations occurring in more than one study cohort (*FANCD2* c.2715 + 1G > A, *TEX15 *c.8325G > A and *RNF168* c.640_644del5) were performed using logistic regression model combining all the analyzed cohorts and exploiting all individual datasets. All the analyses were performed using IBM SPSS Statistics 22.0 for Windows (SPSS Inc., Chicago, IL, USA). All *p*-values were two-sided and values <0.05 were considered statistically significant.

## Electronic supplementary material

Supplementary tables S1-S6, Supplementary figures S1 and S2
