# Supplementary material for: Case-control analysis of truncating mutations in DNA damage response genes connects TEX15 and FANCD2 with hereditary breast cancer susceptibility
Source: Sci Rep. 2017 Apr 6;7:681. doi: 10.1038/s41598-017-00766-9 (PMC5429682; doi:10.1038/s41598-017-00766-9)
Supplement: Supplementary file 1 — Supplementary tables S1-S6, Supplementary figures S1 and S2 [file 41598_2017_766_MOESM1_ESM.pdf]

Supplementary information for

Case-control analysis of truncating mutations in DNA damage response genes connects *TEX15* and *FANCD2* with hereditary breast cancer susceptibility

Tuomo Mantere[#], Anna Tervasmäki[#], Anna Nurmi, Katrin Rapakko, Saila Kauppila, Jiangbo Tang, Johanna Schleutker, Anne Kallioniemi, Jaana M. Hartikainen, Arto Mannermaa, Pentti Nieminen, Riitta Hanhisalo, Sini Lehto, Maija Suvanto, Mervi Grip, Arja Jukkola-Vuorinen, Maria Tengström, Päivi Auvinen, Anders Kvist, Åke Borg, Carl Blomqvist, Kristiina Aittomäki, Roger A. Greenberg, Robert Winqvist*, Heli Nevanlinna & Katri Pylkäs*

[#] Equal contribution

* Correspondence to:

Dr. Katri Pylkäs, Laboratory of Cancer Genetics and Tumor Biology, Biocenter Oulu,

P.O. Box 5000, FI-90014 University of Oulu, Oulu, Finland

katri.pylkas@oulu.fi +358-8-3153650

Professor Robert Winqvist, Laboratory of Cancer Genetics and Tumor Biology, Biocenter Oulu,

P.O. Box 5000, FI-90014 University of Oulu, Oulu, Finland

robert.winqvist@oulu.fi +358-8-3153228

Supplementary table S1. Prevalence of 39 mutations in DNA damage response genes in the Northern Finnish case-control cohorts

Mutation	Cohort	N	WT (%)	Carrier (%)	OR	95% CI	p [a]	OMIM[b]
APTX	Hereditary BC	243	240 (98.8)	3[d] (1.2)	2.344	0.389-14.130	0.385	*606350
c.185-2A>G	Unselected BC	342	342 (100)	0 (ND)	NA	NA	NA	
rs150506419	All BC	585	582 (99.5)	3[d] (0.5)	0.966	0.161-5.811	1.000	
	Controls	377	375 (99.5)	2 (0.5)				
BRE	Hereditary BC	237	236 (99.6)	1 (0.4)	1.589	0.099-25.526	1.000	
c.1089-2A>C	Unselected BC	341	341 (100)	0 (ND)	NA	NA	1.000	
rs150302537	All BC	578	577 (99.8)	1 (0.2)	0.650	0.041-10.422	1.000	
	Controls	376	375 (99.7)	1 (0.3)				
CEP164	Hereditary BC	247	243 (98.4)	4 (1.6)	1.383	0.387-4.945	0.736	*614848
c.1410-2A>G	Unselected BC	651	640 (98.3)	11 (1.7)	1.444	0.530-3.931	0.624	
rs200074826	All BC	898	883 (98.3)	15 (1.7)	1.427	0.550-3.701	0.504	
	Controls	510	504 (98.8)	6 (1.2)				
CHD1L	Hereditary BC	247	246 (99.6)	1 (0.4)	NA	NA	0.378	
c.1386-2A>G	Unselected BC	429	429 (100)	0 (ND)	NA	NA	NA	
rs113139670	All BC	676	675 (99.9)	1 (0.1)	NA	NA	1.000	
	Controls	407	407 (100)	0 (ND)				
CHEK1	Hereditary BC	247	245 (99.2)	2 (0.8)	2.043	0.372-11.217	0.338	
c.289+1G>T	Unselected BC	1173	1170 (99.7)	3 (0.3)	0.642	0.143-2.874	0.710	
Glu97Ter	All BC	1420	1415 (99.6)	5 (0.4)	0.884	0.237-3.301	1.000	
	Controls	1005	1001 (99.6)	4 (0.4)				
CHEK2	Hereditary BC	247	246 (99.6)	1[e] (0.4)	2.236	0.139-35.890	0.524	
c.319+2T>A	Unselected BC	651	651 (100)	0 (ND)	NA	NA	0.458	
	All BC	898	897 (99.9)	1[e] (0.1)	0.613	0.038-9.823	1.000	
	Controls	551	550 (99.8)	1 (0.2)				
CNTLN	Hereditary BC	247	243 (98.4)	4 (1.6)	1.218	0.324-4.581	0.746	
c.1087C>T	Unselected BC	432	427 (98.8)	5 (1.2)	0.867	0.249-3.017	1.000	
Gln363Ter	All BC	679	670 (98.7)	9 (1.3)	0.994	0.331-2.988	1.000	
rs185810944	Controls	375	370 (98.7)	5 (1.3)				
CYP19A1	Hereditary BC	243	242 (99.6)	1 (0.4)	NA	NA	0.391	*107910
c.858+1G>A	Unselected BC	353	353 (100)	0 (ND)	NA	NA	NA	
	All BC	596	595 (99.8)	1 (0.2)	NA	NA	1.000	
	Controls	378	378 (100)	0 (ND)				
DCLRE1A	Hereditary BC	247	246 (99.6)	1 (0.4)	0.373	0.042-3.384	0.653	
c.412C>T	Unselected BC	352	349 (99.1)	3 (0.9)	0.795	0.177-3.578	1.000	
Arg138Ter	All BC	599	595 (99.3)	4 (0.7)	0.622	0.155-2.502	0.492	
rs41292634	Controls	374	370 (98.9)	4 (1.1)				
DCLRE1A	Hereditary BC	247	246 (99.6)	1 (0.4)	NA	NA	0.398	
c.301_302delAG	Unselected BC	352	352 (100)	0 (ND)	NA	NA	NA	
Leu101fs	All BC	599	598 (99.8)	1 (0.2)	NA	NA	1.000	
	Controls	374	374 (100)	0 (ND)				
EIF3K	Hereditary BC	243	242 (99.6)	1 (0.4)	0.775	0.070-8.591	1.000	
c.415C>T	Unselected BC	651	649 (99.7)	2 (0.3)	0.578	0.081-4.119	0.627	
Arg139Ter	All BC	894	891 (99.7)	3 (0.3)	0.631	0.105-3.794	0.637	
rs759176086	Controls	377	375 (99.5)	2 (0.5)				

EME2	Hereditary BC	246	242 (98.4)	4[e] (1.6)	2.022	0.449-9.114	0.446	
c.1159C>T	Unselected BC	514	512 (99.6)	2 (0.4)	0.478	0.079-2.874	0.655	
Gln387Ter	All BC	760	754 (99.2)	6[e] (0.8)	0.973	0.242-3.914	1.000	
rs61753375	Controls	370	367 (99.2)	3 (0.8)				
ENDOV	Hereditary BC	247	239 (96.8)	8[d] (3.2)	1.502	0.556-4.057	0.447	
c.385C>T	Unselected BC	344	340 (98.8)	4 (1.2)	0.528	0.158-1.769	0.387	
Arg129Ter	All BC	591	579 (98.0)	12[d] (2.0)	0.930	0.377-2.297	1.000	
rs199843685	Controls	367	359 (97.8)	8 (2.2)				
ERCC2	Hereditary BC	247	246 (99.6)	1 (0.4)	NA	NA	0.307	*126340
c.2167C>T	Unselected BC	432	432 (100)	0 (ND)	NA	NA	NA	
Gln723Ter	All BC	679	678 (99.9)	1 (0.1)	NA	NA	1.000	
rs754313108	Controls	558	558 (100)	0 (ND)				
ERCC6	Hereditary BC	247	246 (99.6)	1 (0.4)	1.024	0.106-9.893	1.000	*609413
c.3862C>T	Unselected BC	654	652 (99.7)	2 (0.3)	0.773	0.129-4.640	1.000	
Arg1288Ter	All BC	901	898 (99.7)	3 (0.3)	0.842	0.169-4.183	1.000	
rs185142838	Controls	759	756 (99.6)	3 (0.4)				
EXO1	Hereditary BC	247	246 (99.6)	1 (0.4)	NA	NA	0.360	
c.1201C>T	Unselected BC	426	426 (100)	0 (ND)	NA	NA	NA	
Arg401Ter	All BC	673	672 (99.9)	1 (0.1)	NA	NA	1.000	
rs764668398	Controls	439	439 (100)	0 (ND)				
EXO1	Hereditary BC	247	246 (99.6)	1 (0.4)	0.269	0.035-2.097	0.313	
c.2152C>T	Unselected BC	818	807 (98.7)	11 (1.3)	0.903	0.389-2.096	0.833	
Gln718Ter	All BC	1065	1053 (98.9)	12 (1.1)	0.755	0.331-1.721	0.527	
rs146594026	Controls	740	729 (98.5)	11 (1.5)				
FAM83A	Hereditary BC	247	242 (98.0)	5 (2.0)	2.946	0.927-9.361	0.068	
c.1039C>T	Unselected BC	1152	1145 (99.4)	7 (0.6)	0.872	0.305-2.493	1.000	
Arg347Ter	All BC	1399	1387 (99.1)	12 (0.9)	1.233	0.484-3.144	0.816	
rs201762275	Controls	1005	998 (99.3)	7 (0.7)				
FANCD2	Hereditary BC	247	244 (98.8)	3[c] (1.2)	7.537	1.253-45.344	0.036	
c.2715+1G>A	Unselected BC	1152	1150 (99.8)	2 (0.2)	1.066	0.150-7.581	1.000	
E906LfsX4	All BC	1399	1394 (99.6)	5[c] (0.4)	2.199	0.426-11.353	0.459	
rs201811817	Controls	1228	1226 (99.8)	2 (0.2)				
FANCM	Hereditary BC	247	242 (98.0)	5 (2.0)	1.150	0.381-3.469	0.780	
c.5101C>T	Unselected BC	1151	1134 (98.5)	17 (1.5)	0.835	0.369-1.885	0.671	
Q1701X	All BC	1398	1376 (98.4)	22 (1.6)	0.890	0.407-1.946	0.838	
rs147021911	Controls	510	501 (98.2)	9 (1.8)				
FANCM	Hereditary BC	247	244 (98.8)	3 (1.2)	2.078	0.416-10.370	0.398	
c.5791C>T	Unselected BC	1151	1145 (99.5)	6 (0.5)	0.886	0.221-3.555	1.000	
R1931X	All BC	1398	1389 (99.4)	9 (0.6)	1.095	0.295-4.061	1.000	
rs144567652	Controls	510	507 (99.4)	3 (0.6)				
GNL3	Hereditary BC	239	238 (99.6)	1 (0.4)	NA	NA	0.402	
c.1611_1612insA	Unselected BC	342	342 (100)	0 (ND)	NA	NA	NA	
Glu537ArgfsTer3	All BC	581	580 (99.8)	1 (0.2)	NA	NA	1.000	
	Controls	355	355 (100)	0 (ND)				
IGHMBP2	Hereditary BC	247	246 (99.6)	1 (0.4)	NA	NA	0.398	*600502
c.1488C>A	Unselected BC	342	342 (100)	0 (ND)	NA	NA	NA	
Cys496Ter	All BC	589	588 (99.8)	1 (0.2)	NA	NA	1.000	
rs145226920	Controls	373	373 (100)	0 (ND)				

INO80C	Hereditary BC	247	245 (99.2)	2 (0.8)	3.837	0.346-42.523	0.273	
c.563_564insC	Unselected BC	498	497 (99.8)	1 (0.2)	0.946	0.059-15.162	1.000	
Thr188fs	All BC	745	742 (99.6)	3 (0.4)	1.900	0.197-18.322	1.000	
	Controls	471	470 (99.8)	1 (0.2)				
MSH3	Hereditary BC	247	246 (99.6)	1 (0.4)	NA	NA	0.300	
c.697G>T	Unselected BC	1237	1236 (99.9)	1 (0.1)	NA	NA	NA	
Glu233Ter	All BC	1484	1482 (99.9)	2 (0.1)	NA	NA	1.000	
rs756632960	Controls	575	575 (100)	0 (ND)				
NAT10	Hereditary BC	240	239 (99.6)	1 (0.4)	NA	NA	0.390	
c.1224delG	Unselected BC	429	429 (100)	0 (ND)	NA	NA	NA	
Trp408fs	All BC	669	668 (99.9)	1 (0.1)	NA	NA	1.000	
	Controls	375	375 (100)	0 (ND)				
NEIL1	Hereditary BC	245	244 (99.6)	1[e] (0.4)	1.340	0.121-14.846	1.000	
c.314dupC	Unselected BC	1116	1107 (99.2)	9 (0.8)	2.659	0.573-12.342	0.229	
Pro106AlafsTer50	All BC	1361	1351 (99.3)	10[e] (0.7)	2.420	0.529-11.078	0.357	
rs556576971	Controls	656	654 (99.7)	2 (0.3)				
NINL	Hereditary BC	239	238 (0.6)	1 (0.4)	NA	NA	0.431	
c.2446C>T	Unselected BC	605	604 (99.8)	1 (0.2)	NA	NA	1.000	
Arg816Ter	All BC	844	842 (99.8)	2 (0.2)	NA	NA	1.000	
rs145851710	Controls	410	410 (100)	0 (ND)				
NTHL1	Hereditary BC	243	241 (99.2)	2 (0.8)	0.391	0.081-1.900	0.316	*602656
c.268C>T	Unselected BC	342	340 (99.4)	2 (0.6)	0.277	0.057-1.345	0.105	
Gln90Ter	All BC	585	581 (99.3)	4 (0.7)	0.325	0.094-1.117	0.110	
rs150766139	Controls	337	330 (97.9)	7 (2.1)				
POLQ	Hereditary BC	247	246 (99.6)	1 (0.4)	0.912	0.106-7.843	1.000	
c.4262_4268del7	Unselected BC	1148	1141 (99.4)	7 (0.6)	1.377	0.436-4.350	0.774	
Ile1421ArgfsTer8	All BC	1395	1387 (99.4)	8 (0.6)	1.294	0.422-3.967	0.783	
rs546221341	Controls	1127	1122 (99.6)	5 (0.4)				
PTPRH	Hereditary BC	247	246 (99.6)	1 (0.4)	NA	NA	0.398	
c.3190C>T	Unselected BC	344	344 (100)	0 (ND)	NA	NA	NA	
Gln1064Ter	All BC	591	590 (99.8)	1 (0.2)	NA	NA	1.000	
rs768918412	Controls	374	374 (100)	0 (ND)				
RNF168	Hereditary BC	247	243 (98.4)	4[c] (1.6)	3.235	0.906-11.549	0.077	*612688
c.640_644del5	Unselected BC	1194	1185 (99.2)	9 (0.8)	1.492	0.530-4.206	0.606	
Lys214Terfs	All BC	1441	1428 (99.1)	13[c] (0.9)	1.789	0.678-4.721	0.257	
rs777601326	Controls	1185	1179 (99.5)	6 (0.5)				
SPP1	Hereditary BC	240	239 (99.6)	1 (0.4)	1.573	0.098-25.271	1.000	
c.94-1G>A	Unselected BC	342	342 (100)	0 (ND)	NA	NA	1.000	
rs139555315	All BC	582	581 (99.8)	1 (0.2)	0.647	0.040-10.378	1.000	
	Controls	377	376 (99.7)	1 (0.3)				
TEX15	Hereditary BC	247	240 (97.2)	7[d] (2.8)	2.670	1.054-6.762	0.063	
c.8325G>A	Unselected BC	1318	1308 (99.2)	10 (0.8)	0.700	0.306-1.602	0.411	
Trp2775Ter	All BC	1565	1548 (98.9)	17[d] (1.1)	1.005	0.486-2.078	1.000	
rs146619272	Controls	1203	1190 (98.9)	13 (1.1)				
TEX15	Hereditary BC	247	244 (98.8)	3 (1.2)	14.619	1.514-141.129	0.018	
c.7253dupT	Unselected BC	1317	1314 (99.8)	3 (0.2)	2.715	0.282-26.132	0.627	
Leu2418PhefsTer6	All BC	1564	1558 (99.6)	6 (0.4)	4.579	0.551-38.085	0.149	
rs760604179	Controls	1190	1189 (99.9)	1 (0.1)				

TOP3A	Hereditary BC	247	246 (99.6)	1 (0.4)	NA	NA	0.397	
c.403C>T	Unselected BC	520	520 (100)	0 (ND)	NA	NA	NA	
Arg135Ter	All BC	767	766 (99.9)	1 (0.1)	NA	NA	1.000	
rs200944917	Controls	375	375 (100)	0 (ND)				
TUBGCP5	Hereditary BC	246	245 (99.6)	1 (0.4)	0.759	0.068-8.418	1.000	
c.1813C>T	Unselected BC	344	342 (99.4)	2 (0.6)	1.088	0.152-7.764	1.000	
Arg605Ter	All BC	590	587 (99.5)	3 (0.5)	0.951	0.158-5.716	1.000	
rs145962755	Controls	374	372 (99.5)	2 (0.5)				
UVSSA	Hereditary BC	240	238 (99.2)	2 (0.8)	3.118	0.281-34.572	0.564	*614632
c.405C>G	Unselected BC	429	429 (100)	0 (ND)	NA	NA	0.464	
Tyr135Ter	All BC	669	667 (99.7)	2 (0.3)	1.112	0.101-12.309	1.000	
rs770825185	Controls	372	371 (99.7)	1 (0.3)				
ZRANB3	Hereditary BC	236	235 (99.6)	1 (0.4)	NA	NA	0.386	
c.2336_2337delAA	Unselected BC	344	343 (99.7)	1 (0.3)	NA	NA	0.478	
Lys779ThrfsTer15	All BC	580	578 (99.7)	2 (0.3)	NA	NA	0.522	
rs531232289	Controls	376	376 (100)	0 (ND)				

[a] *p* -value of χ2 test or Fisher's exact test

[b] OMIM number for the genes associated with a hereditary syndrome

[c] Includes one *BRCA1* mutation carrier

[d] Includes one *BRCA2* mutation carrier

[e] Includes one homozygote carrier

BC: breast cancer, CI: confidence interval, Mut: mutation carrier, NA: not analyzed, ND: not detected, OR: odds ratio, WT: wild type.

Supplementary table S2. Tumor characteristics of *TEX15* c.7253dupT and *FANCD2* c.2715+1G>A mutation carriers

Mutation	Family[a] / sample ID	Cohort	Histology	ER	PR	HER2	Grade	Ki-67	TNM	Age at diagnosis
TEX15										
c.7253dupT	Family 1	Oulu hereditary	DCIS	0	0	1	NA	1	T1N0M0	39
	Family 2	Oulu hereditary	DCIS	NA	NA	NA	NA	NA	T?N0M0	36
	Family 3	Oulu hereditary	Ductal	1	1	0	2	3	T1N0M0	38
	Family 4	Oulu unselected	Ductal	1	1	1	3	3	NA	37
	Family 5	Oulu unselected	Ductal	1	1	0	1	1	NA	58
	Family 6	Oulu unselected	NEBC	1	1	0	NA	1	NA	47
FANCD2										
c.2715+1G>A	Family 1	Oulu hereditary	Ductal	0	0	0	3	3	T2N0M0	39
	Family 2	Oulu hereditary	Ductal	1	1	1	3	3	T3N2M0	40
	Family 4	Helsinki Hereditary	Ductal	0	0	0	3	3	T2N1M0	41
	Family 5	Helsinki Hereditary	Ductal	NA	NA	NA	2	NA	T?N?M0	44
	Family 6	Helsinki Hereditary/ unselected	Lobular	0	0	0	2	0	T2N1M0	49
	Family 7	Helsinki Hereditary	Ductal	1	1	0	NA	1	T1N0M0	68
	Case A	Oulu unselected	Ductal	1	1	0	3	1	T2N1M0	48
	Case B	Oulu unselected	Ductal	1	1	0	1	1	T1N1M0	66
	Case C	Helsinki Unselected	Ductal	1	1	0	1	0	T1N0M0	62
	Case D	Helsinki Unselected	Ductal	1	0	0	2	2	T1N1M0	64
	Case E	Helsinki Unselected	Tubular	1	1	0	1	1	T1N0M0	51

DCIS: ductal carcinoma in situ, ER: estrogen receptor, HER2: Human epidermal growth factor receptor 2, M: primary metastasis, N: nodal status, NEBC: neuroendocrine breast carcinoma, PR: progesterone receptor, T: tumor size

1=positive, 0=negative

[a] Supplementary figure S1

Supplementary table S3. Occurrence of chromosomal aberrations in blood lymphocyte metaphases of heterozygous *TEX15* c.7253dupT mutation carriers and wild type controls

**Type of aberration**	**Median of chromosomal aberrations observed per 100 metaphases (min – max)**		***p*-value[a]**
	**Carriers (n =6)**	**Controls (n = 9)**	
Telomeric associations	ND	ND	1.0
Chromatid/chromosome breaks, deletions	1.0 (0.0 – 6.0)	1.8 (0.0 – 4.0)	0.933
Simple chromosomal rearrangements[b]	2.0 (0 – 6.0)	0.0 (0.0 – 2.0)	0.094
Complex chromosomal rearrangements[c]	0.0 (0.0 – 4.0)	ND	0.143
Total rearrangements (simple+complex)	2.0 (0 – 10.0)	0.0 (0.0 – 2.0)	0.067

[a] Mann-Whitney U-test

[b] Inversions, ring chromosomes, translocations (≤3 break rearrangements)

[c] Translocations (≥4 break rearrangements) and marker chromosomes

ND: not detected

Supplementary table S4. 796 sequenced DNA damage response genes, their genomic interval, number of regions, target size and coverage

Target ID	Interval (Hg19)	Number of sequenced regions	Size (bp)	Coverage (%)
AATF	chr17:35306164-35414181	12	2381	99.58
ABCE1	chr4:146019073-146050686	15	6910	98.70
ABCF2	chr7:150904912-150924327	18	4809	99.98
ABL1	chr9:133589257-133763072	14	6642	97.82
ACACA	chr17:35441912-35766912	63	12644	99.89
ACTL6A	chr3:179280657-179306206	14	3955	99.44
ACTR5	chr20:37377074-37401099	9	2671	100.00
ACTR8	chr3:53901082-53916239	14	4346	99.86
AFAP1	chr4:7760429-7941663	21	9736	100.00
AHR	chr7:17338235-17385786	11	6881	99.14
AIFM1	chrX:129263326-129299871	17	3646	98.77
AKAP8	chr19:15464321-15490622	14	4079	100.00
AKAP8L	chr19:15490848-15529843	14	2394	100.00
AKT1	chr14:105235675-105262098	9	11018	98.17
ALKBH1	chr14:78138736-78174373	7	2984	99.66
ALKBH2	chr12:109525982-109531446	3	2350	100.00
ALKBH3	chr11:43902346-43941835	12	2625	99.28
ANKLE1	chr19:17392443-17398465	9	3398	97.82
AP1B1	chr22:29723658-29819178	24	7201	99.38
AP2A1	chr19:50270169-50310379	24	3985	100.00
AP2A2	chr11:924883-1012255	28	15702	98.45
AP2B1	chr17:33914271-34053446	25	6567	100.00
AP3B2	chr15:83328022-83378676	29	8094	99.84
AP5S1	chr20:3801160-3805964	3	2020	96.58
AP5Z1	chr7:4815242-4834036	14	7236	98.66
APBB1	chr11:6416343-6440654	14	6826	100.00
APEX1	chr14:20923279-20925943	3	2299	98.17
APEX2	chrX:55026745-55035500	6	3399	100.00
APITD1	chr1:10490148-10512220	7	3039	99.34
APLF	chr2:68694680-68858014	19	5753	98.84
APTX	chr9:32972593-33025176	13	4052	100.00
AR	chrX:66763863-66950471	12	13001	99.92
AREG	chr4:75310840-75490495	12	3126	44.27
ARF1	chr1:228270350-228286923	8	3441	99.07
ARF4	chr3:57557079-57583957	8	2755	99.13
ARHGEF2	chr1:155916619-155966139	33	8438	99.91
ARIH1	chr15:72766656-72879702	16	7566	99.41
ARL13A	chrX:100224686-100245830	9	1717	100.00
ARL2	chr11:64781574-64789667	5	2185	96.93
ASCC3	chr6:100956059-101329258	43	12591	98.84
ASF1A	chr6:119215230-119230345	4	2745	99.42
ASTE1	chr3:130732708-130746503	10	3627	99.97
ATF2	chr2:175936967-176033120	21	6171	99.27
ATM	chr11:108093200-108239839	64	23604	98.03
ATMIN	chr16:81069441-81080973	5	6362	98.98
ATP5A1	chr18:43664099-43684310	14	3162	99.15

ATR	chr3:142168066-142297678	47	13436	99.43
ATRIP	chr3:48488103-48507125	14	3697	99.95
ATRX	chrX:76760345-77041729	38	13017	99.59
ATXN3	chr14:92524885-92572975	21	9299	95.66
AURKA	chr20:54944434-54967403	11	3148	98.51
AURKB	chr17:8108038-8113954	8	2429	99.55
AXIN2	chr17:63524670-63557775	15	5496	99.55
BABAM1	chr19:17378221-17390172	10	1718	100.00
BAG2	chr6:57037093-57050022	5	2371	100.00
BAG5	chr14:104022870-104029178	3	5331	99.49
BAP1	chr3:52435014-52444376	15	5359	99.44
BARD1	chr2:215590359-215674438	13	6322	99.73
BAT1	chr6:31497985-31510235	9	7187	99.11
BAX	chr19:49458061-49465065	3	2666	98.69
BAZ1B	chr7:72854717-72936625	19	7440	99.88
BCCIP	chr10:127512093-127542274	9	4693	99.55
BIRC5	chr17:76210266-76221726	6	2844	99.86
BLM	chr15:91260547-91358869	24	6510	98.53
BMI1	chr10:22605370-22620424	12	4199	96.33
BMS1	chr10:43277943-43330395	23	8508	94.16
BRAP	chr12:112079939-112123800	12	4926	99.90
BRCA1	chr17:41196301-41322300	26	8817	97.30
BRCA2	chr13:32889600-32973819	29	12251	98.11
BRCC3	chrX:154299684-154351359	12	3422	99.36
BRE	chr2:28112797-28561778	18	3804	99.45
BRIP1	chr17:59756536-59940930	24	10589	97.09
BRIX1	chr5:34915470-34926111	5	5111	93.05
BSG	chr19:571266-583503	11	3896	97.36
BTG2	chr1:203274608-203278740	2	2797	100.00
BUB1	chr2:111395264-111435701	29	6622	100.00
BUB1B	chr15:40453199-40513388	25	5057	99.60
C11ORF30	chr11:76155956-76264079	22	12223	98.01
C17ORF70	chr17:79506900-79520997	12	5620	99.18
C19ORF40	chr19:33463137-33467970	5	1238	96.45
C1ORF57	chr1:233086340-233114558	7	10914	99.73
C1ORF86	chr1:2115888-2144169	15	8450	97.98
CALU	chr7:128379335-128413487	9	6545	100.00
CAND1	chr12:67663050-67713741	18	14032	98.97
CASP3	chr4:185548839-185570673	9	3079	100.00
CAV1	chr7:116164828-116201249	4	4163	99.76
CCNA1	chr13:37005956-37017029	10	2502	99.64
CCNA2	chr4:122737588-122745098	8	2958	99.43
CCNB1	chr5:68462826-68474082	9	2738	100.00
CCND1	chr11:69455844-69469252	5	4930	99.80
CCNE1	chr19:30302794-30315226	11	2495	99.76
CCNH	chr5:86687300-86708860	9	4854	98.93
CCNO	chr5:54526969-54529518	3	1869	100.00
CCT3	chr1:156278741-156337674	18	4385	99.93
CCT6A	chr7:56119312-56131692	15	4201	99.12
CD2BP2	chr16:30362076-30366692	6	3893	99.82

CDC14B	chr9:99252512-99382122	21	10877	98.57
CDC25A	chr3:48198625-48229902	15	4430	100.00
CDC25B	chr20:3767567-3786772	17	4489	100.00
CDC25C	chr5:137620943-137674054	14	3335	100.00
CDC45	chr22:19466971-19508145	20	3246	100.00
CDC6	chr17:38443874-38459423	12	3864	98.68
CDC73	chr1:193091077-193223952	19	6702	96.33
CDCA5	chr11:64833761-64851646	8	4085	100.00
CDCA8	chr1:38158062-38175401	10	2686	99.63
CDH13	chr16:82660388-83830225	27	7719	99.26
CDK1	chr10:62538078-62554620	9	4089	99.49
CDK2	chr12:56360542-56366578	6	4014	99.53
CDK4	chr12:58141499-58149806	10	3368	98.60
CDK7	chr5:68530611-68573267	13	2676	99.59
CDK9	chr9:130547947-130553076	6	3129	99.42
CDKN1A	chr6:36644226-36655126	7	3034	99.74
CDKN1B	chr12:12867981-12875315	5	2911	99.69
CDKN2A	chr9:21967740-21995310	9	4618	99.11
CDKN2D	chr19:10677127-10679665	2	1463	100.00
CDS2	chr20:5107396-5178543	14	12198	99.44
CEBPG	chr19:33864564-33873602	3	3920	99.74
CEP164	chr11:117185262-117283994	37	13202	99.95
CEP170	chr1:243287719-243418718	25	11497	71.29
CEP192	chr18:12991350-13125061	49	13588	99.65
CETN2	chrX:151995506-151999331	4	2008	100.00
CHAF1A	chr19:4402648-4443404	15	3644	99.56
CHAF1B	chr21:37757665-37789135	16	3386	99.23
CHD1L	chr1:146714280-146767457	24	3854	98.75
CHD4	chr12:6679237-6716652	40	8275	99.82
CHEK1	chr11:125495020-125546160	14	6846	98.96
CHEK2	chr22:29083720-29138420	23	4605	90.60
CHRNA4	chr20:61974651-62009763	12	9764	99.81
CIB1	chr15:90773196-90777289	7	1387	100.00
CINP	chr14:102808945-102829263	11	3748	91.65
CIRH1A	chr16:69165183-69202951	20	5797	99.98
CLSPN	chr1:36185808-36235578	28	10167	98.88
CNTLN	chr9:17134969-17503931	29	11747	98.04
COBRA1	chr9:140149614-140168010	13	2960	100.00
CPS1	chr2:211342395-211543841	44	11344	99.05
CRB2	chr9:126118437-126142613	15	6650	99.85
CREB1	chr2:208394450-208470294	15	12317	98.99
CREBBP	chr16:3775044-3930737	31	15688	99.77
CRY1	chr12:107385131-107487645	14	5370	99.63
CRY2	chr11:45868658-45904809	14	6480	99.98
CSNK1D	chr17:80196888-80231617	15	11342	90.46
CSNK1E	chr22:38686686-38794537	22	7139	98.61
CSTF1	chr20:54967416-54979592	6	2908	99.76
CSTF2	chrX:100075337-100096495	16	3947	100.00
CTBP1	chr4:1205217-1243751	16	8689	89.88
CTCFL	chr20:56071010-56100718	15	8250	98.16

CUL4A	chr13:113862541-113919409	24	6264	99.36
CUL4B	chrX:119658435-119709694	26	7866	98.39
CYP19A1	chr15:51500243-51630817	19	8835	99.76
CYP1A1	chr15:75011872-75017961	7	3575	99.97
DAPK1	chr9:90112132-90323559	30	9583	99.71
DBF4	chr7:87505520-87538866	12	4823	99.36
DCLRE1A	chr10:115594472-115614173	10	4951	98.08
DCLRE1B	chr1:114447752-114456718	4	4020	100.00
DCLRE1C	chr10:14939347-14996441	20	7733	99.75
DCP2	chr5:112312388-112357902	12	11422	98.65
DDB1	chr11:61066908-61110078	28	14514	99.75
DDB2	chr11:47236482-47260779	10	2048	99.51
DDR1	chr6:30844187-30867943	25	9627	99.77
DDX1	chr2:15731291-15771245	26	4337	98.96
DDX19B	chr16:70323555-70369196	16	4842	98.51
DDX23	chr12:49223528-49246635	17	5778	98.39
DDX39A	chr19:14519599-14530205	14	2099	98.86
DDX39B	chr6:31497985-31510262	9	7369	99.13
DDX6	chr11:118618462-118661982	15	11744	97.39
DEK	chr6:18224088-18265064	12	4601	98.15
DEM1	chr1:40974402-40982238	2	3132	98.56
DGCR8	chr22:20067744-20099410	12	7555	100.00
DHX9	chr1:182808428-182857127	31	6581	99.51
DICER1	chr14:95552554-95624357	32	13276	99.53
DKC1	chrX:153991020-154005974	15	4489	99.89
DMC1	chr22:38914943-38966301	16	3844	98.73
DNA2	chr10:70173810-70231889	22	5446	99.94
DNAJA1	chr9:33025198-33039915	10	2930	98.60
DNAJA2	chr16:46989263-47007709	8	5224	98.35
DNAJA3	chr16:4475795-4506786	13	7331	98.13
DNAJB11	chr3:186285181-186315071	12	6280	99.79
DNAJC10	chr2:183580757-183653552	27	7934	99.36
DNAJC7	chr17:40128428-40169725	15	2504	100.00
DROSHA	chr5:31400591-31532313	41	8606	99.72
DSP	chr6:7541797-7586960	25	10605	99.59
DTL	chr1:212208908-212280752	16	5212	99.41
DYRK2	chr12:68042107-68059196	6	9614	99.74
E2F1	chr20:32263281-32274220	7	2840	99.26
E2F2	chr1:23832909-23857722	7	5599	99.82
E2F4	chr16:67226057-67232831	6	3706	100.00
E2F6	chr2:11584490-11606307	9	4241	90.71
EBNA1BP2	chr1:43629834-43727599	15	3831	99.56
ECEL1	chr2:233344526-233352548	17	3312	99.64
EEPD1	chr7:36192747-36341162	8	5007	99.78
EGFR	chr7:55086703-55324323	36	13691	99.51
EID3	chr12:104697499-104698992	1	1493	100.00
EIF2C2	chr8:141541253-141645728	22	5264	99.62
EIF3A	chr10:120794345-120840406	22	6792	95.30
EIF3E	chr8:109213434-109447572	23	6465	99.41
EIF3G	chr19:10225679-10230609	11	1347	99.55

EIF3H	chr8:117654358-117779174	15	6731	98.35
EIF3I	chr1:32687518-32697215	11	1945	97.94
EIF3K	chr19:39109711-39127605	8	1406	99.93
EIF3L	chr22:38244864-38285424	15	4643	97.85
EIF4E	chr4:99792824-99851798	11	14289	94.64
EIF4EBP1	chr8:37887848-37917893	4	1153	100.00
EME1	chr17:48450570-48458854	7	3434	99.48
EME2	chr16:1823197-1831719	7	7112	99.89
ENDOV	chr17:78388954-78411896	17	7621	97.90
EP300	chr22:41487779-41576091	31	10205	99.33
EPC2	chr2:149401998-149545146	17	5921	99.63
ERBB2	chr17:37844156-37886689	31	10941	98.91
ERCC1	chr19:45910580-45982096	12	4151	99.69
ERCC2	chr19:45854235-45873886	23	3913	99.64
ERCC3	chr2:128014855-128051762	12	7643	99.96
ERCC4	chr16:14014003-14046215	13	8724	96.29
ERCC5	chr13:103459694-103528361	27	8682	98.68
ERCC6	chr10:50663403-50747594	27	11594	99.20
ERCC8	chr5:60169647-60240915	17	5192	97.00
ESCO1	chr18:19109231-19180855	15	5588	93.04
ESCO2	chr8:27629455-27670167	16	8293	95.92
ESR1	chr6:151977815-152450764	18	13990	98.88
ETFA	chr15:76507685-76603823	17	5371	96.85
ETS1	chr11:128328645-128457463	13	6432	99.19
ETS2	chr21:40177220-40196889	13	4855	99.79
ETV7	chr6:36321987-36356174	9	3011	98.27
EWSR1	chr22:29663987-29696525	15	10628	98.17
EXO1	chr1:242011258-242058460	19	4520	97.96
EXOSC10	chr1:11126664-11159948	23	6463	99.29
EXOSC9	chr4:122722461-122738186	12	4951	98.87
EYA1	chr8:72109657-72274477	19	5633	99.56
EYA2	chr20:45523252-45817502	20	4022	100.00
EYA3	chr1:28296844-28415217	20	6883	99.45
EYA4	chr6:133561725-133853268	28	9528	99.85
FAM175A	chr4:84382081-84444511	13	6302	99.03
FAM83A	chr8:124191189-124222328	7	6456	99.91
FAN1	chr15:31196044-31235321	16	6514	99.65
FANCA	chr16:89803946-89883075	41	10790	98.31
FANCB	chrX:14861518-14891201	10	3415	99.88
FANCC	chr9:97861325-98080001	22	8383	99.62
FANCD2	chr3:10068087-10143624	46	9298	97.14
FANCE	chr6:35420127-35434891	10	2755	100.00
FANCF	chr11:22644068-22647397	1	3329	99.76
FANCG	chr9:35073821-35080023	12	3854	99.61
FANCI	chr15:89787169-89860502	41	8589	98.95
FANCL	chr2:58386367-58468525	14	2715	99.78
FANCM	chr14:45605125-45670103	25	8975	99.83
FBXL7	chr5:15500294-15939910	5	4949	99.82
FBXO18	chr10:5931524-5979568	29	7427	99.77
FBXO6	chr1:11724139-11734421	7	1982	99.45

FEN1	chr11:61560098-61564726	2	2518	98.53
FGF10	chr5:44303635-44389818	4	3091	98.19
FGFR2	chr10:123237833-123357982	31	8425	98.94
FHIT	chr3:59735025-61237143	17	5779	98.62
FHL2	chr2:105974158-106055240	10	6622	95.70
FLNA	chrX:153576881-153603016	41	11405	100.00
FOS	chr14:75745466-75748947	3	3301	100.00
FOXA1	chr14:38058746-38069255	5	4643	100.00
FOXM1	chr12:2966836-2986331	10	5099	100.00
FTO	chr16:53737864-54155863	16	13043	99.27
FZR1	chr19:3506284-3536765	14	3904	99.62
GADD45A	chr1:68150733-68154031	3	2129	100.00
GADD45G	chr9:92219916-92221480	3	1383	98.77
GANAB	chr11:62392287-62414114	25	5433	98.36
GEN1	chr2:17935114-17966642	16	6979	98.93
GLUD1	chr10:88809948-88854786	19	4862	99.73
GNL3	chr3:52715161-52728520	14	3600	99.86
GNL3L	chrX:54556633-54593730	17	9145	97.77
GPR3	chr1:27719137-27722328	2	2155	100.00
GSTCD	chr4:106629924-106768895	14	6584	98.28
GSTP1	chr11:67351055-67354141	3	1985	100.00
GTF2H1	chr11:18343805-18388601	17	6379	99.59
GTF2H2	chr5:70330773-70363526	18	7609	17.86
GTF2H2C	chr5:68856024-68890560	19	7476	22.39
GTF2H3	chr12:124118364-124146489	14	3513	97.87
GTF2H4	chr6:30875950-30881893	12	2593	99.54
GTF2H5	chr6:158589368-158620386	3	7546	98.24
GTPBP4	chr10:1034327-1065886	17	6501	95.66
H2AFX	chr11:118964553-118966187	1	1634	100.00
HADHA	chr2:26413493-26467604	20	4371	96.84
HADHB	chr2:26466027-26513346	18	4640	99.63
HDAC1	chr1:32757676-32799246	13	4269	99.39
HDAC2	chr6:114254181-114332482	16	15797	98.16
HERC2	chr15:28356172-28567308	93	21890	87.59
HIC1	chr17:1957437-1962991	2	4594	99.67
HINFP	chr11:118992222-119006762	9	10195	96.66
HK1	chr10:71029729-71161648	30	6766	100.00
HK2	chr2:75059771-75120496	19	7770	98.35
HMG20B	chr19:3572764-3579096	6	3977	99.07
HMGA1	chr6:34204566-34214018	7	2715	85.75
HMGA2	chr12:66217900-66360085	10	15008	97.73
HMGB1	chr13:31032868-31191744	7	5974	86.49
HMGB2	chr4:174252516-174256286	3	3251	100.00
HMGN1	chr21:40714230-40721583	5	5202	94.08
HNRNPR	chr1:23630253-23670863	14	8980	98.32
HNRNPU	chr1:245013591-245027854	13	9618	98.91
HPDL	chr1:45792534-45794357	1	1823	100.00
HSPA4	chr5:132387643-132442151	21	5552	100.00
HSPA5	chr9:127997116-128003676	8	4130	96.22
HSPB1	chr7:75931850-75933624	2	1676	99.88

HSPD1	chr2:198351294-198381471	14	4525	95.25
HUS1	chr7:47735317-48019232	12	4865	98.46
HUS1B	chr6:655928-656974	1	1046	99.04
HUWE1	chrX:53559046-53713707	87	19143	99.69
ID4	chr6:19837590-19842441	3	3936	99.92
IDH3A	chr15:78423829-78464301	16	7152	99.13
IDH3B	chr20:2639030-2644875	8	2729	100.00
IFI16	chr1:158969747-159024955	14	5574	94.53
IFNB1	chr9:21077093-21077972	1	879	100.00
IGF1	chr12:102789634-102874433	7	8227	99.68
IGHMBP2	chr11:68671299-68708080	17	6805	99.99
IKBKG	chrX:153769403-153796792	15	7762	28.96
ILF2	chr1:153634253-153643534	13	2541	97.28
ILF3	chr19:10764926-10803105	21	7899	100.00
INCENP	chr11:61891434-61920645	19	4853	100.00
INIP	chr9:115448775-115480526	7	2070	94.11
INO80	chr15:41267878-41408562	38	10963	99.82
INO80B	chr2:74682139-74688021	8	2833	99.75
INO80C	chr18:33034775-33077965	8	1395	96.70
INO80D	chr2:206858434-206951037	12	14613	99.80
INO80E	chr16:30006604-30017124	6	6196	100.00
INTS3	chr1:153700532-153746565	26	10241	98.31
IRS1	chr2:227596022-227664485	3	10013	98.77
JAK1	chr1:65298895-65432197	27	6863	99.85
JAK2	chr9:4985022-5128193	27	6608	99.14
JMJD1C	chr10:64926970-65225732	28	11663	99.07
JMY	chr5:78531914-78623048	11	9349	99.22
JUN	chr1:59246449-59250009	1	3560	100.00
KAT2B	chr3:20081504-20195906	19	6014	99.97
KAT5	chr11:65479456-65487087	13	3994	99.60
KBTBD2	chr7:32907767-32933753	8	5153	98.58
KCNH6	chr17:61600684-61626348	15	4511	99.69
KEAP1	chr19:10596785-10614064	7	2841	100.00
KHDRBS1	chr1:32479419-32526461	11	3808	99.74
KIAA0101	chr15:64657182-64679896	7	2642	94.32
KIAA1524	chr3:108268707-108308501	22	5012	99.22
KIAA1543	chr19:7660777-7683204	19	4563	99.28
KIAA2022	chrX:73952673-74145330	4	11839	99.65
KIF22	chr16:29802023-29816716	10	4518	99.54
KIF2A	chr5:61601978-61833086	29	6156	99.68
KIN	chr10:7792914-7830000	14	7789	93.04
KLK3	chr19:51358160-51364030	5	2314	98.36
KPNA1	chr3:122140737-122233802	18	8859	99.31
KPNA2	chr17:66031624-66042980	12	3392	97.91
KPNB1	chr17:45726831-45762881	27	8218	95.34
KRAS	chr12:25357712-25403880	7	7442	97.47
KRT5	chr12:52908348-52914481	7	4440	99.50
LIG1	chr19:48618692-48673611	29	4784	99.00
LIG3	chr17:33307506-33332098	17	7438	97.98
LIG4	chr13:108859781-108870726	4	4487	99.89

LIMD1	chr3:45596875-45727840	11	12893	97.07
LMNA	chr1:156052353-156109890	22	7425	99.89
LMO4	chr1:87794140-87814617	6	5954	99.45
LMO7	chr13:76194559-76434016	36	12989	99.92
LTB	chr6:31548291-31550309	3	1595	100.00
MAD2L2	chr1:11734526-11751717	7	3544	99.72
MAGED2	chrX:54834021-54842455	13	3174	99.81
MAP3K1	chr5:56110889-56191989	19	8597	100.00
MAP7	chr6:136663408-136871967	22	5926	99.49
MBD2	chr18:51677960-51751168	10	6013	99.93
MBD3	chr19:1576667-1592720	6	2737	100.00
MBD4	chr3:129149776-129158888	7	3352	99.70
MC1R	chr16:89984276-89987395	1	3119	99.84
MCM7	chr7:99690340-99699573	13	4647	98.17
MCM8	chr20:5931287-5975862	20	4320	96.92
MCM9	chr6:119134601-119256337	14	12796	99.74
MCPH1	chr8:6264102-6501154	18	6106	99.23
MCRS1	chr12:49950316-49961946	15	3563	100.00
MDC1	chr6:30667573-30685676	13	8393	99.38
MDM2	chr12:69201945-69239330	15	8438	97.87
MDM4	chr1:204485496-204542881	17	11729	94.82
MED17	chr11:93517382-93547871	13	11033	97.81
MED21	chr12:27175444-27219286	9	3836	99.90
MEN1	chr11:64570975-64578776	9	4422	100.00
MGMT	chr10:131265437-131566281	9	3737	99.20
MLH1	chr3:37034812-37107390	24	4234	98.91
MLH3	chr14:75480456-75518245	15	8663	99.15
MMS19	chr10:99218070-99258561	28	6421	99.97
MMS22L	chr6:97590026-97731103	25	22293	98.31
MNAT1	chr14:61201448-61436681	9	4046	100.00
MND1	chr4:154265790-154336280	11	1733	99.37
MOGS	chr2:74688173-74692547	3	3471	100.00
MORF4	chr4:174537076-174538067	1	991	100.00
MORF4L1	chr15:79102818-79190485	18	6944	98.99
MORF4L2	chrX:102930413-102943096	6	4813	99.71
MPG	chr16:126995-135862	6	1781	98.99
MRE11A	chr11:94150458-94227084	22	6404	99.78
MSH2	chr2:47630097-47789460	20	4999	99.08
MSH3	chr5:79950456-80172644	26	5584	99.50
MSH4	chr1:76262545-76378933	20	3677	99.97
MSH5	chr6:31707714-31732638	28	7932	94.39
MSH6	chr2:48010210-48034102	14	5535	96.28
MTDH	chr8:98656396-98742498	12	8121	99.61
MTFR1	chr8:66556113-66683506	17	5139	99.61
MUM1	chr19:1285879-1378440	17	4756	99.96
MUS81	chr11:65624586-65635134	11	5703	99.79
MUTYH	chr1:45794824-45806152	11	4238	99.95
MYBBP1A	chr17:4442180-4458936	23	6697	99.73
MYC	chr8:128747669-128753690	3	3061	100.00
NABP1	chr2:192542783-192553261	7	5816	98.35

NABP2	chr12:56615788-56623648	7	2358	95.93
NAT10	chr11:34127100-34169227	32	5920	99.61
NBN	chr8:90945553-91015466	22	6681	99.19
NBR1	chr17:41322487-41363717	24	5674	99.95
NCOA2	chr8:71021986-71316050	26	10662	99.51
NCOA3	chr20:46130590-46285631	25	8949	99.79
NCOA6	chr20:33302567-33413462	17	10202	98.09
NDNL2	chr15:29560342-29562043	1	1701	97.77
NDUFA9	chr12:4714099-4798464	16	5894	98.81
NDUFS1	chr2:206986138-207024337	20	6060	96.65
NDUFS3	chr11:47586877-47606125	7	3811	100.00
NEIL1	chr15:75639285-75647602	10	5275	99.89
NEIL2	chr8:11627133-11644865	5	3473	99.48
NEIL3	chr4:178230979-178284107	10	3159	97.69
NF1	chr17:29421934-29709144	62	27930	98.97
NFKB1	chr4:103422475-103538469	29	5782	99.83
NFRKB	chr11:129733659-129765500	31	6821	99.77
NHEJ1	chr2:219940028-220025597	13	3369	100.00
NINL	chr20:25433327-25566163	24	6491	99.72
NME1	chr17:49230886-49239799	5	3540	99.04
NOL11	chr17:65713938-65740657	18	5073	99.05
NOL6	chr9:33461340-33473951	25	6532	100.00
NOLC1	chr10:103911922-103923637	13	4226	100.00
NONO	chrX:70503031-70521028	16	5646	97.86
NPM1	chr5:170814109-170838151	11	4048	99.51
NR1H2	chr19:50832938-50886295	14	2968	100.00
NR2E3	chr15:72084966-72110610	11	3723	100.00
NSMCE1	chr16:27236301-27280125	13	4030	99.21
NSMCE2	chr8:126103910-126379377	14	2999	99.63
NSMCE4A	chr10:123716592-123734753	10	3757	99.36
NTHL1	chr16:2089805-2097877	8	2590	99.69
NUDT1	chr7:2281846-2290791	6	2128	99.48
NUDT16	chr3:131100504-131107684	1	7180	99.36
NUFIP1	chr13:45513373-45563628	10	3692	93.09
OBFC2A	chr2:192542783-192553261	7	5816	98.35
OBFC2B	chr12:56615788-56623648	7	2358	95.93
OGG1	chr3:9791617-9829913	10	4222	99.76
OPTC	chr1:203463260-203478087	8	1604	100.00
OTUB1	chr11:63753314-63769293	8	5496	93.50
P4HA1	chr10:74766964-74856742	17	3526	98.78
P4HA2	chr5:131527520-131631018	23	8594	99.65
P4HB	chr17:79801023-79818580	10	5505	100.00
PALB2	chr16:23614472-23652688	15	5092	99.65
PAPD7	chr5:6714707-6757171	13	5447	99.94
PARG	chr10:51026314-51371341	19	5366	70.15
PARP1	chr1:224102730-226595811	25	7750	99.21
PARP2	chr14:20811730-20826074	14	4376	99.82
PARP3	chr3:51976350-51982893	10	3128	100.00
PARP4	chr13:24995053-25086958	34	6760	94.33
PARPBP	chr12:102513945-102591308	16	5180	99.52

PAXIP1	chr7:154735386-154794804	24	10128	99.94
PC	chr11:66615693-66725857	28	6487	99.85
PCNA	chr20:5095588-5107282	7	1611	98.14
PDCD4	chr10:112631542-112659774	14	4827	95.17
PDLIM4	chr5:131593340-131609157	7	3345	100.00
PFKL	chr21:45719914-45747271	20	9337	99.12
PFKM	chr12:48498911-48540197	31	7942	99.28
PFKP	chr10:3109701-3179914	28	5309	100.00
PGM3	chr6:83870858-83903665	17	8148	100.00
PGR	chr11:100900344-101001265	9	15486	99.33
PIAS1	chr15:68346506-68483106	22	4964	99.66
PIAS4	chr19:4007737-4039393	11	3274	98.84
PKP3	chr11:392603-404918	15	4087	99.54
PLAT	chr8:42032225-42065252	15	7449	96.29
PLK1	chr16:23688966-23701698	9	4940	100.00
PLK3	chr1:45265886-45271677	11	3704	99.11
PMS1	chr2:190648800-190742365	17	5794	99.29
PMS2	chr7:6012859-6048766	14	4216	73.79
PNKP	chr19:50364449-50370832	17	2061	100.00
POLA1	chrX:24712025-25015113	38	7096	99.90
POLB	chr8:42195961-42229341	18	4471	99.73
POLD1	chr19:50887569-50921285	27	4007	99.78
POLD2	chr7:44154268-44163967	12	3949	100.00
POLD3	chr11:74204885-74380172	16	5835	99.83
POLD4	chr11:67118225-67124453	2	3102	94.04
POLE	chr12:133200334-133413397	44	17808	99.17
POLE2	chr14:50110259-50155150	21	3231	97.99
POLG	chr15:89859523-89878102	22	5971	99.93
POLG2	chr17:62473891-62493194	12	3658	100.00
POLH	chr6:43543867-43588270	11	8632	92.49
POLI	chr18:51795763-51847646	14	9754	99.02
POLK	chr5:74807570-74896979	17	9445	98.93
POLL	chr10:103338628-103348037	8	5894	100.00
POLM	chr7:44111835-44122149	11	4707	99.92
POLN	chr4:2073634-2243858	32	8381	99.58
POLQ	chr3:121150262-121265498	32	10036	99.34
POLR2A	chr17:7387674-7417945	28	8915	98.62
POLR2B	chr4:57843877-57897344	26	6421	99.72
POLR2C	chr16:57496288-57505932	7	4080	100.00
POLR2D	chr2:128603829-128615741	5	2611	99.96
POLR2E	chr19:1086567-1095401	8	3026	99.14
POLR2F	chr22:38348603-38437123	14	5565	98.89
POLR2G	chr11:62529000-62534197	6	2053	100.00
POLR2H	chr3:184079495-184086394	7	3286	99.15
POLR2I	chr19:36604600-36606216	6	992	100.00
POLR2J	chr7:102113537-102119391	4	1086	76.61
POLR2K	chr8:101162801-101166240	3	1771	100.00
POLR2L	chr11:837345-842555	3	1202	100.00
POU2F1	chr1:167190055-167396592	27	16334	99.00
POU4F1	chr13:79173216-79177705	2	3857	99.71

POU4F2	chr4:147560034-147563636	2	3184	99.69
PPM1D	chr17:58677533-58743650	7	5028	94.81
PPP1CA	chr11:67165641-67188664	8	2873	100.00
PPP1R9B	chr17:48211090-48228001	10	4493	99.51
PPP2CA	chr5:133530014-133561960	10	5978	98.88
PPP2R5A	chr1:212458771-212535215	19	4248	99.69
PPP2R5B	chr11:64685014-64701960	18	3770	100.00
PPP2R5C	chr14:102228124-102394338	29	14622	97.95
PPP2R5D	chr6:42952226-42980093	14	3615	100.00
PPP2R5E	chr14:63838064-64010102	15	9488	98.40
PPP4C	chr16:30087288-30096708	6	2854	100.00
PPP4R2	chr3:73045925-73118360	12	6207	93.57
PRKCG	chr19:54382433-54410916	21	3847	99.77
PRKDC	chr8:48685658-48872753	86	17144	99.49
PRMT6	chr1:107599256-107601926	1	2670	100.00
PRPF19	chr11:60658009-60674071	17	3262	99.85
PRPF31	chr19:54618779-54635160	12	2924	100.00
PRPF6	chr20:62612420-62664463	21	3521	100.00
PRPH	chr12:49687024-49692491	8	4530	99.78
PSMC1	chr14:90722828-90738978	9	3536	94.65
PSMC2	chr7:102984690-103009852	14	3818	98.98
PSMC3	chr11:47440309-47448034	11	2498	99.72
PSMC6	chr14:53173879-53195315	12	7722	96.90
PSMD1	chr2:231921567-232037551	26	5691	99.95
PSMD14	chr2:162164538-162268238	12	7938	99.53
PSMD2	chr3:184016486-184026852	15	5761	98.68
PSMD3	chr17:38137010-38154223	12	5241	99.81
PTEN	chr10:89622859-89731697	10	10248	97.20
PTPRH	chr19:55692604-55720884	20	4328	99.49
PTTG1	chr5:159848818-159855758	4	2102	100.00
RAD1	chr5:34905355-34919104	10	6967	96.10
RAD17	chr5:68665109-68710640	22	4602	97.13
RAD18	chr3:8817077-9005467	15	7283	100.00
RAD21	chr8:117858162-117887115	14	6473	98.36
RAD21L1	chr20:1206689-1277075	17	3070	99.48
RAD23A	chr19:13056617-13064467	9	2001	99.00
RAD23B	chr9:110045407-110094485	12	4855	99.30
RAD50	chr5:131891700-131980323	29	8671	99.38
RAD51	chr15:40986961-41024366	13	3139	99.65
RAD51AP1	chr12:4647939-4669224	12	2812	100.00
RAD51AP2	chr2:17691840-17699716	3	3784	99.21
RAD51B	chr14:68286485-69196945	33	8164	98.73
RAD51C	chr17:56769923-56811713	11	3569	99.92
RAD51D	chr17:33426800-33448551	14	3355	100.00
RAD52	chr12:1021232-1100366	15	5255	99.71
RAD54B	chr8:95384177-95487353	19	9605	98.73
RAD54L	chr1:46713349-46744155	18	3572	100.00
RAD54L2	chr3:51575585-51697622	20	6597	99.15
RAD9A	chr11:67159165-67165893	10	3377	96.36
RAD9B	chr12:110939449-110969901	14	3458	99.91

RAN	chr12:131356413-131362233	3	4885	97.79
RASGRF1	chr15:79252278-79383225	31	10649	99.97
RASSF1	chr3:50367206-50378421	7	3819	98.80
RAVER1	chr19:10426878-10444324	12	3921	99.97
RB1	chr13:48877872-49056132	28	6733	99.50
RBBP4	chr1:33116732-33151822	15	9771	94.34
RBBP7	chrX:16857395-16888547	11	7612	97.24
RBBP8	chr18:20378213-20606461	29	5673	99.58
RBL1	chr20:35624741-35724420	23	6426	97.82
RBL2	chr16:53467878-53525571	27	8070	99.67
RBM14	chr11:66384042-66413948	12	8899	99.64
RBX1	chr22:41347340-41369323	5	3192	100.00
RCC1	chr1:28832444-28865718	19	4165	99.88
RCHY1	chr4:76404236-76439984	7	6705	99.79
RCL1	chr9:4792823-4861074	12	3049	100.00
RCN2	chr15:77223949-77242611	7	6027	98.62
RDM1	chr17:34245059-34257790	6	2098	95.81
RECQL	chr12:21621833-21654613	15	4079	94.61
RECQL4	chr8:145736656-145743239	19	4646	97.91
RECQL5	chr17:73622914-73663279	22	9206	99.89
RELA	chr11:65421056-65430575	7	5604	99.55
REV1	chr2:100016927-100106507	25	8269	99.75
REV3L	chr6:111620223-111804928	37	13193	98.30
RFC1	chr4:39289058-39368011	32	7368	98.76
RFC2	chr7:73645818-73668784	13	2461	99.31
RFC3	chr13:34392175-34540705	10	3085	100.00
RFC4	chr3:186507658-186524857	11	3217	98.79
RFC5	chr12:118451382-118470945	15	3557	100.00
RFWD2	chr1:175911237-176176639	25	4956	98.39
RFWD3	chr16:74655281-74700789	16	6785	99.72
RHEB	chr7:151163087-151217216	10	3735	97.59
RHNO1	chr12:2985413-2998701	5	2564	99.65
RIF1	chr2:152266386-152364537	46	14393	99.53
RIOK1	chr6:7389718-7418280	16	3557	99.72
RNF11	chr1:51701932-51739137	3	3182	99.59
RNF144B	chr6:18368768-18469115	10	5646	99.27
RNF168	chr3:196195643-196230649	6	5467	93.98
RNF169	chr11:74459902-74553468	6	7943	99.60
RNF2	chr1:185014485-185071750	8	4233	99.57
RNF20	chr9:104296122-104325636	20	5094	99.74
RNF8	chr6:37321737-37362524	11	6795	99.15
RNMTL1	chr17:685502-695759	4	2145	99.91
RPA1	chr17:1732985-1803386	20	5892	99.13
RPA2	chr1:28218024-28241267	8	2179	99.27
RPA3	chr7:7676138-7758248	8	3399	100.00
RPA4	chrX:96138896-96140476	1	1580	100.00
RPAIN	chr17:5322950-5336350	5	7674	97.71
RPL10L	chr14:47120202-47121038	1	836	100.00
RPL15	chr3:23958025-23965197	2	6451	96.16
RPL18A	chr19:17970676-17974972	5	1600	100.00

RPL27A	chr11:8703947-8736316	4	7273	99.01
RPL37A	chr2:217362901-217443913	2	5663	99.75
RPL5	chr1:93297583-93307491	8	3499	99.11
RPL7A	chr9:136215058-136218291	6	2362	100.00
RPS2	chr16:2012042-2014871	2	2453	95.84
RPS27A	chr2:55459028-55462999	4	3210	100.00
RPS27L	chr15:63418060-63450230	4	4117	100.00
RPS3	chr11:75110519-75133355	8	6075	99.74
RPS5	chr19:58898625-58906181	6	861	99.42
RRAD	chr16:66955571-66959557	4	1789	100.00
RRM2B	chr8:103216718-103251356	10	5778	98.34
RTEL1	chr20:62289152-62330061	36	10949	99.97
RUVBL1	chr3:127783610-127872767	13	7127	99.17
RUVBL2	chr19:49497145-49519192	15	2798	100.00
SART1	chr11:65729149-65747617	18	4993	99.12
SCRIB	chr8:144873079-144897559	38	6952	99.96
SDF4	chr1:1152277-1167457	7	4873	100.00
SDHA	chr5:218345-256825	16	8870	89.43
SET	chr9:131445692-131458689	13	4642	92.91
SETMAR	chr3:4344977-4359261	4	3647	97.37
SETX	chr9:135136732-135230382	29	12926	99.49
SFPQ	chr1:35641968-35658759	14	7706	98.86
SFR1	chr10:105881805-105886153	4	2620	100.00
SHFM1	chr7:96110927-96339213	16	7928	99.63
SHPRH	chr6:146185370-146285569	33	18297	97.40
SIRT1	chr10:69644416-69678157	11	4771	99.90
SIRT6	chr19:4174095-4182606	8	1796	98.55
SKIV2L2	chr5:54603565-54721419	28	7150	98.63
SLC25A5	chrX:118602352-118605369	5	1677	99.46
SLC30A9	chr4:41992478-42089561	20	4702	100.00
SLK	chr10:105726948-105789001	19	8354	99.89
SLX1A	chr16:29465811-30208897	10	3883	13.44
SLX1B	chr16:29465811-30208897	10	3837	13.60
SLX4	chr16:3631171-3661609	14	8871	99.31
SMAD3	chr15:67356090-67487543	18	9473	100.00
SMAD4	chr18:48494399-48611425	16	9489	99.59
SMARCA1	chrX:128580467-128657487	25	4603	99.87
SMARCA2	chr9:2015331-2193634	43	7992	99.87
SMARCA4	chr19:11071587-11172968	41	6887	99.75
SMARCA5	chr4:144434605-144478652	24	8868	99.88
SMARCAD1	chr4:95128748-95212453	28	6892	99.38
SMARCD2	chr17:61909430-61920435	15	4085	99.61
SMC1A	chrX:53401059-53449687	26	11203	100.00
SMC1B	chr22:45739933-45809510	25	4753	98.95
SMC2	chr9:106856530-106903710	27	7033	99.70
SMC3	chr10:112327438-112364404	30	4875	98.30
SMC4	chr3:160117051-160152760	23	8935	99.75
SMC5	chr9:72873867-72969814	25	7098	97.25
SMC6	chr2:17845068-17981519	35	10063	98.30
SMG1	chr16:18816164-18937786	64	26507	87.44

SMUG1	chr12:54558518-54582788	8	5500	99.75
SMURF2	chr17:62538402-62658396	22	7601	99.36
SND1	chr7:127292191-127732671	35	7232	100.00
SNRNP40	chr1:31732404-31769672	11	4233	98.51
SOD1	chr21:33031924-33041254	6	2139	100.00
SP1	chr12:53773949-53810240	7	8223	97.76
SPO11	chr20:55904804-55919060	14	2147	99.77
SPP1	chr4:88896791-88904573	8	2466	97.49
SPRTN	chr1:231472839-231490779	6	6026	99.85
SRBD1	chr2:45615808-45839314	25	5545	99.69
SSRP1	chr11:57093448-57103361	11	6543	99.77
STAT1	chr2:191829073-191885696	31	6682	99.63
STAT5A	chr17:40439554-40463971	21	5359	96.40
STRA13	chr17:79976567-79981993	3	1591	99.06
STUB1	chr16:730104-732809	4	2441	98.53
SUB1	chr5:32531728-32604195	10	9524	98.64
SUCLA2	chr13:48510611-48612135	17	4127	97.99
SUGT1	chr13:53226820-53262443	15	2214	99.01
SUMO1	chr2:203070892-203103341	9	2345	96.16
SUN2	chr22:39130708-39190158	22	6960	99.71
SUPT16H	chr14:21819620-21852435	28	7741	97.29
SUPT6H	chr17:26989098-27029707	37	11355	99.96
SWI5	chr9:131037647-131051279	7	1789	100.00
SWSAP1	chr19:11485372-11487637	2	1702	95.36
SYNCRIP	chr6:86267685-86353520	19	9249	98.63
TACC1	chr8:38585693-38710556	23	13069	99.71
TAOK1	chr17:27717471-27878932	20	17364	97.61
TAOK2	chr16:29984951-30003592	19	9445	99.97
TAOK3	chr12:118587595-118810760	28	11130	99.50
TAPBP	chr6:33267460-33282174	6	5176	95.94
TARBP2	chr12:53894694-53900225	7	4230	100.00
TBL3	chr16:2022027-2032944	19	7519	99.73
TCEA1	chr8:54879101-54935099	13	4653	93.68
TCHP	chr12:110338058-110421656	18	9414	99.33
TCOF1	chr5:149737191-149779881	23	12117	99.88
TCP1	chr6:160199519-160210791	10	5157	99.32
TDG	chr12:104359571-104382666	11	5127	73.45
TDP1	chr14:90421272-90511118	25	6725	99.36
TDP2	chr6:24650194-24667271	7	3222	98.11
TELO2	chr16:1543334-1560470	18	5010	99.80
TERF1	chr8:73921086-73960367	12	9082	90.75
TERF2	chr16:69389453-69442484	12	4464	99.89
TERF2IP	chr16:75681624-75795061	5	2820	99.93
TEX15	chr8:30689049-30748132	13	11923	98.65
TFDP1	chr13:114238992-114295798	15	4080	97.08
TFDP2	chr3:141663259-141868396	20	12335	97.29
TFPT	chr19:54610309-54619065	7	1380	100.00
TICRR	chr15:90118807-90171263	22	7109	99.92
TIMM50	chr19:39971041-39981538	11	2792	98.07
TIPARP	chr3:156391013-156424569	11	5268	100.00

TMEM161A	chr19:19229967-19249320	12	2514	99.16
TNP1	chr2:217724170-217724797	2	447	100.00
TONSL	chr8:145654152-145669837	21	7500	98.17
TOP1	chr20:39657447-39753137	21	4158	98.44
TOP2A	chr17:38544757-38574418	44	7938	99.60
TOP3A	chr17:18174731-18218331	22	7087	99.94
TOPBP1	chr3:133317008-133380747	28	7075	99.00
TOX3	chr16:52471906-52581724	11	5251	99.81
TP53	chr17:7565086-7590878	14	4216	94.83
TP53BP1	chr15:43699396-43802936	33	9503	99.69
TP73	chr1:3569073-3652775	16	5887	99.93
TPX2	chr20:30326893-30389618	19	4155	99.47
TRAF7	chr16:2205688-2228140	22	4929	99.63
TREX1	chr3:48506434-48509054	2	2237	100.00
TREX2	chrX:152710167-152736055	14	2995	100.00
TRIM28	chr19:59055825-59062092	17	3299	100.00
TRIM29	chr11:119981972-120056247	19	10598	99.84
TRIM40	chr6:30103874-30116522	6	2615	98.36
TRIP12	chr2:230631919-230787965	47	10485	99.47
TRIP13	chr5:892747-919482	15	3860	100.00
TSC1	chr9:135766724-135820030	24	10393	99.86
TSHR	chr14:81421322-81612656	11	5805	99.97
TSPAN17	chr5:176074377-176086069	6	5339	100.00
TSR1	chr17:2225940-2240811	15	5734	94.91
TTC5	chr14:20724706-20774163	14	9985	99.24
TUBB3	chr16:89987789-90005179	7	4718	97.73
TUBB6	chr18:12308059-12326578	3	2399	100.00
TUBG1	chr17:40761347-40767266	11	2171	95.07
TUBGCP4	chr15:43661408-43699303	20	8895	98.91
TUBGCP5	chr15:22833384-22873902	24	6420	99.22
TUBGCP6	chr22:50656107-50683431	22	8129	98.57
TUFM	chr16:28853721-28857739	9	2451	100.00
TWIST1	chr7:19060603-19157305	6	2153	99.54
TXNIP	chr1:145438451-145442645	5	3733	100.00
TYMS	chr18:657593-673588	9	2650	99.43
U2AF2	chr19:56165405-56186092	12	3371	99.44
UBA1	chrX:47050188-47074537	29	7105	97.02
UBA52	chr19:18682603-18688280	5	2911	95.12
UBB	chr17:16284101-16286069	3	1681	92.50
UBC	chr12:125396139-125401924	2	3938	76.59
UBE2A	chrX:118708488-118718391	6	3373	100.00
UBE2B	chr5:133706859-133727809	8	3567	99.10
UBE2D1	chr10:60094724-60130523	7	3114	100.00
UBE2D3	chr4:103715529-103790063	12	10370	98.14
UBE2E1	chr3:23847373-23933141	11	3628	100.00
UBE2I	chr16:1355537-1377029	10	12586	96.15
UBE2L3	chr22:21903725-21978333	6	4067	96.90
UBE2N	chr12:93799438-93836048	5	5210	94.78
UBE2T	chr1:202300774-202311118	6	1185	100.00
UBE2V1	chr20:48697650-48732506	16	5247	97.03

UBE2V2	chr8:48920949-48976521	7	5989	97.36
UBE2W	chr8:74650398-74791155	12	9619	99.40
UBE4A	chr11:118230285-118744768	22	7154	99.86
UBE4B	chr1:10092879-10241307	32	9780	99.18
UBR5	chr8:103264490-103425079	58	17810	97.76
UCHL5	chr1:192981485-193029247	15	7292	99.52
UCP2	chr11:73685701-73694362	11	3457	100.00
UHRF1	chr19:4909499-4962175	19	4789	84.34
UIMC1	chr5:176331995-176449644	21	5315	97.48
UNG	chr12:109535368-109548808	8	2728	99.93
UPF1	chr19:18942733-18979049	24	5825	99.90
USP1	chr1:62901957-62917485	10	4463	99.53
USP10	chr16:84733544-84813538	18	5472	99.67
USP28	chr11:113668585-113746302	30	6319	99.78
USP3	chr15:63796699-63886849	23	8224	99.54
USP39	chr2:85829954-85876417	19	4399	100.00
USP44	chr12:95910325-95945276	10	5586	99.23
USP47	chr11:11862959-11980882	31	11780	98.90
USP7	chr16:8985940-9058381	37	8709	99.85
UVRAG	chr11:75526201-75855292	23	7855	97.40
UVSSA	chr4:1341043-1381847	15	8130	98.97
VCP	chr9:35056050-35073256	16	5758	99.57
VEGFA	chr6:43737910-43754234	4	14511	99.79
WDR16	chr17:9479933-9546786	18	3438	99.24
WDR33	chr2:128458585-128568771	23	15095	99.97
WDR48	chr3:39093478-39138165	20	7881	99.52
WEE1	chr11:9595217-9615014	12	9261	97.49
WIZ	chr19:15532307-15560772	14	7714	100.00
WRN	chr8:30890767-31031295	35	6873	98.17
WRNIP1	chr6:2765637-2786937	7	3758	99.92
WWP1	chr8:87354956-87490659	29	6916	98.99
WWP2	chr16:69796176-69975654	34	9917	99.60
XAB2	chr19:7684400-7694461	19	3047	99.70
XPA	chr9:100437180-100459701	8	2045	99.71
XPC	chr3:14186636-14220293	16	5118	99.04
XRCC1	chr19:44047453-44079740	18	2478	99.44
XRCC2	chr7:152341853-152373260	4	4933	93.45
XRCC3	chr14:104163935-104181851	14	7865	99.92
XRCC4	chr5:82373306-82652558	10	2889	98.55
XRCC5	chr2:216972176-217071036	24	5943	99.61
XRCC6	chr22:42017112-42060062	12	3532	99.86
XRCC6BP1	chr12:58335313-58351062	7	1513	100.00
YWHAH	chr22:32340436-32353600	6	2969	99.66
YY1	chr14:100704624-100749139	4	8994	99.89
ZBTB32	chr19:36203819-36207950	7	2122	100.00
ZFYVE26	chr14:68194080-68283317	43	16566	98.76
ZMYND11	chr10:180394-300587	20	6225	99.23
ZNF350	chr19:52467582-52490089	5	2441	94.55
ZRANB3	chr2:135894475-136288816	30	9734	98.53
ZSWIM7	chr17:15879863-15903041	8	2719	97.94

Supplementary table S5. Observed mutations excluded from the case-control analyses

Gene	Mutation*	Disease association [reference]
BRCA1	NM_007294:c.3626delT:p.Leu1209Terfs	Hereditary BC [1]
BRCA1	NM_007294:c.4097-2A>G (rs80358019)	Hereditary BC [2]
BRCA1	NM_007294:c.3718C>T:p.Gln1240Ter (rs80356903)[a]	Hereditary BC [3]
BRCA2	NM_000059:c.9118-2A>G (rs81002862)	Hereditary BC [2]
BRCA2	NM_000059:c.3256_3257insA:p.Ile1086fs[a,b]	Unknown
BRCA2	NM_000059:c.7480C>T:p.Arg2494Ter (rs80358972)	Hereditary BC [2]
BRCA2	NM_000059:c.6275_6276delTT:p.Leu2092Profs (rs11571658)[c]	Hereditary BC [1]
PALB2	NM_024675:c.1592delT:p.Leu531Cysfs	Hereditary BC [4]
ATM	NM_000051:c.6903_6904insA:p.Glu2304GlyfsTer69	Hereditary BC [5]
RAD50	NM_005732:c.687delT:p.Ser229ArgfsTer6	Hereditary BC [6]
RHNO1	NM_001252499:c.C250T:p.Arg84Ter (rs140887418)	No association to hereditary BC [7]
MPG	NM_002434.3:c.40-1G>T (rs146600185)	No association to hereditary BC [8]

* Deleterious mutations in *BRCA1/2* and other mutations previously studied in Finnish population

[a] Previously unreported in the Finnish population

[b] not presented in ExAC or in the Breast Cancer Information Core (BIC) databases

[c] also known as 6503delTT

References

[1] Sarantaus L, Huusko P, Eerola H, Launonen V, Vehmanen P, Rapakko K, et al.
Multiple founder effects and geographical clustering of BRCA1 and BRCA2 families in Finland. *Eur J Hum Genet* 2000;**8**(10):757-763.

[2] Vehmanen P, Friedman LS, Eerola H, McClure M, Ward B, Sarantaus L, et al.
Low proportion of BRCA1 and BRCA2 mutations in Finnish breast cancer families: Evidence for additional susceptibility genes. *Hum Mol Genet* 1997;**6**(13):2309-2315.

[3] Kwon JS, Lenehan J, Carey M, Ainsworth P.
Prolonged survival among women with BRCA germline mutations and advanced endometrial cancer: A case series. *Int J Gynecol Cancer* 2008;**18**(3):546-549.

[4] Erkko H, Xia B, Nikkilä J, Schleutker J, Syrjäkoski K, Mannermaa A, et al.
A recurrent mutation in PALB2 in Finnish cancer families. *Nature* 2007;**446**(7133):316-319.

[5] Pylkäs K, Tommiska J, Syrjäkoski K, Kere J, Gatei M, Waddell N, et al.
Evaluation of the role of Finnish ataxia-telangiectasia mutations in hereditary predisposition to breast cancer. *Carcinogenesis* 2007;**28**(5):1040-1045.

[6] Heikkinen K, Rapakko K, Karppinen S-, Erkko H, Knuutila S, Lundán T, et al.
RAD50 and NBS1 are breast cancer susceptibility genes associated with genomic instability. *Carcinogenesis* 2006;**27**(8):1593-1599.

[7] Heikkinen T, Khan S, Huovari E, Vilske S, Schleutker J, Kallioniemi A, et al.
Evaluation of the RHINO gene for breast cancer predisposition in Finnish breast cancer families. *Breast Cancer Res Treat* 2014;**144**(2):437-441.

[8] Kiiski JI, Pelttari LM, Khan S, Freysteinsdottir ES, Reynisdottir I, Hart SN, et al.
Exome sequencing identifies FANCM as a susceptibility gene for triple-negative breast cancer. *Proc Natl Acad Sci U S A* 2014;**111**(42):15172-15177.

Supplementary table S6. cDNA specific sequencing primers for *TEX15* and *FANCD2* mutations

Primer	Sequence 5' - 3'
*TEX15*_c.8325G>A_cDNA_F	TTTGTGCCAGTGAATGGGTA
*TEX15*_c.8325G>A_cDNA_R	TGGATGAAAGGATTCTTGGTG
*TEX15*_c.7253dupT_cDNA_F	AAGGCAACATTCAAGCATCC
*TEX15*_c.7253dupT_cDNA_R	TCCCATGGTCTGGTGAAAAT
*FANCD2*_c.2715+1G>A_cDNA_F	ACTCTCAACTGGTTCCGAGA
*FANCD2*_c.2715+1G>A_cDNA_R	CCAGCAAGAAAAGCAGCTCA

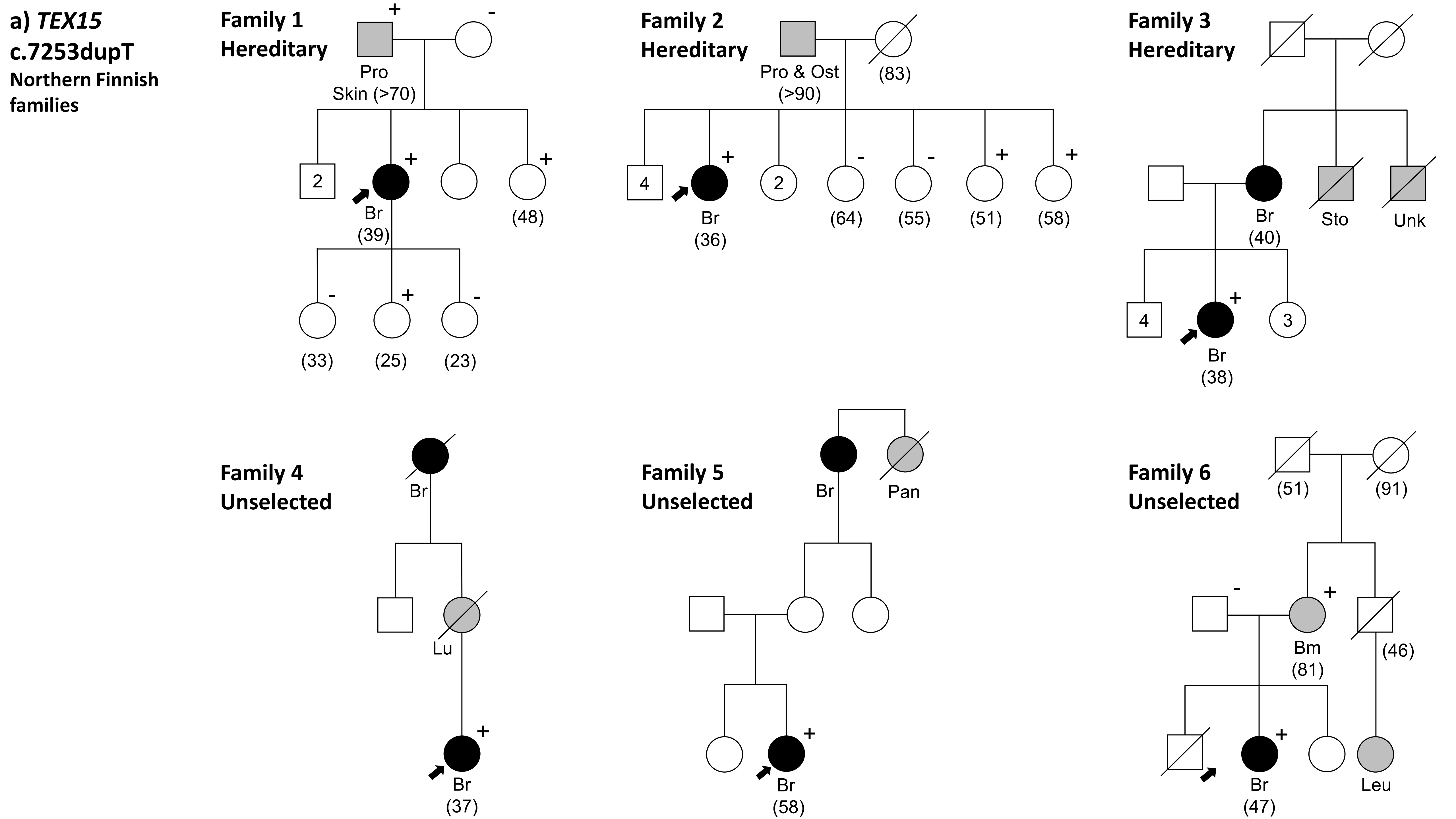
a) *TEX15*
c.7253dupT
Northern Finnish families
Family 1
Hereditary
Pro
Skin (>70)
2
Br
(39)
(48)
(33)
(25)
(23)
Family 2
Hereditary
Pro & Ost
(>90)
(83)
4
Br
(36)
2
(64)
(55)
(51)
(58)
Family 3
Hereditary
Br
(40)
Sto
Unk
4
Br
(38)
3
Family 4
Unselected
Br
Lu
Br
(37)
Family 5
Unselected
Br
Pan
Br
(58)
Family 6
Unselected
(51)
(91)
Bm
(81)
(46)
Br
(47)
Leu

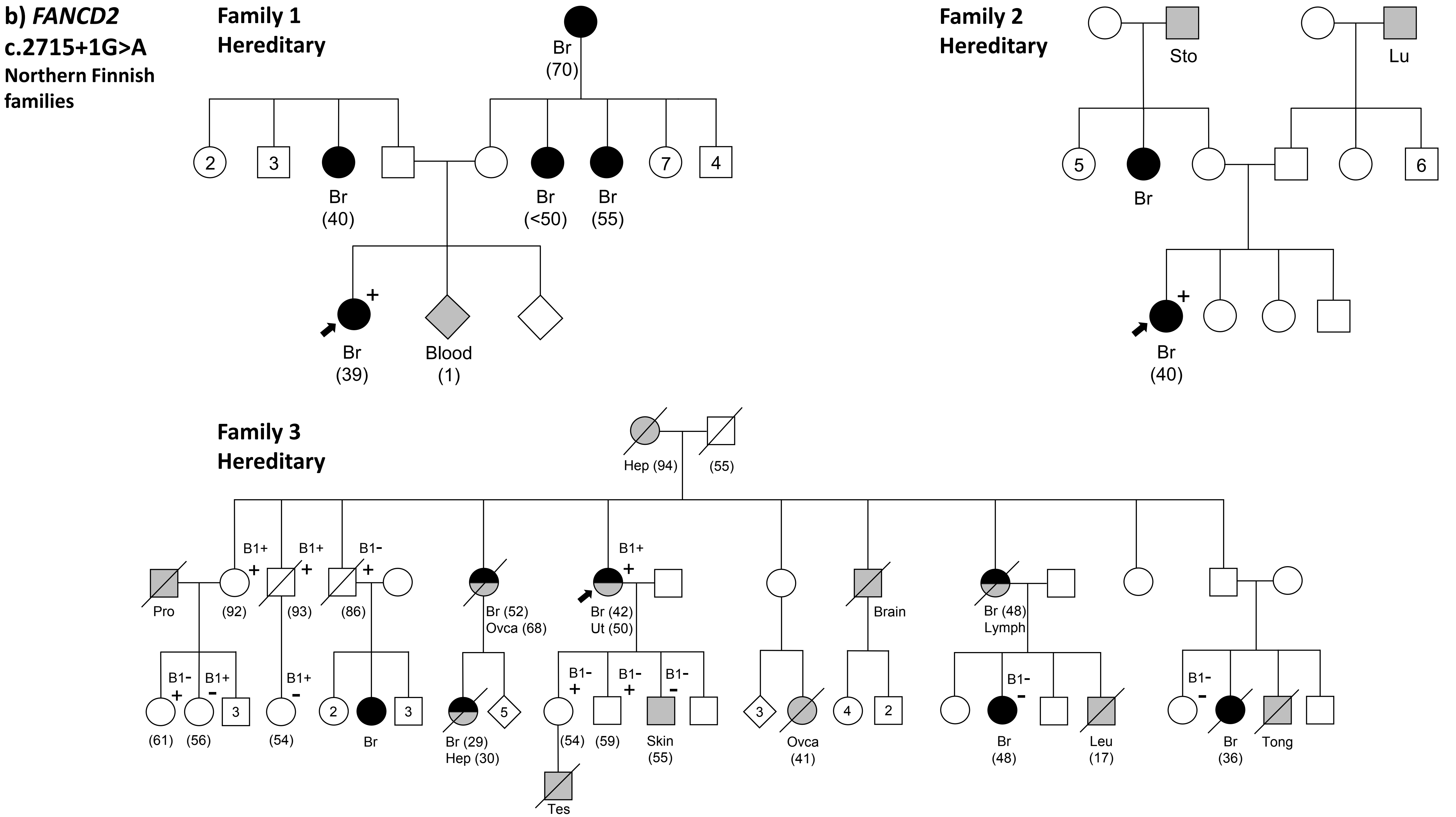
b) FANCD2
c.2715+1G>A
Northern Finnish families
Family 1
Hereditary
Br
(70)
2
3
Br
(40)
Br
(<50)
Br
(55)
7
4
+
Br
(39)
Blood
(1)
Family 2
Hereditary
Sto
Lu
5
Br
6
+
Br
(40)
Family 3
Hereditary
Hep (94)
(55)
B1+
+
B1+
+
B1-
+
B1+
+
Pro
(92)
(93)
(86)
Br (52)
Ovca (68)
Br (42)
Ut (50)
Brain
Br (48)
Lymph
B1-
+
B1+
-
3
(61)
(56)
B1+
-
(54)
2
Br
3
Br (29)
Hep (30)
5
B1-
+
B1-
+
B1-
-
(54)
(59)
Skin
(55)
Tes
3
Ovca
(41)
4
2
B1-
-
Br
(48)
Leu
(17)
B1-
-
Br
(36)
Tong

c) *FANCD2*
c.2715+1G>A
Families from Helsinki

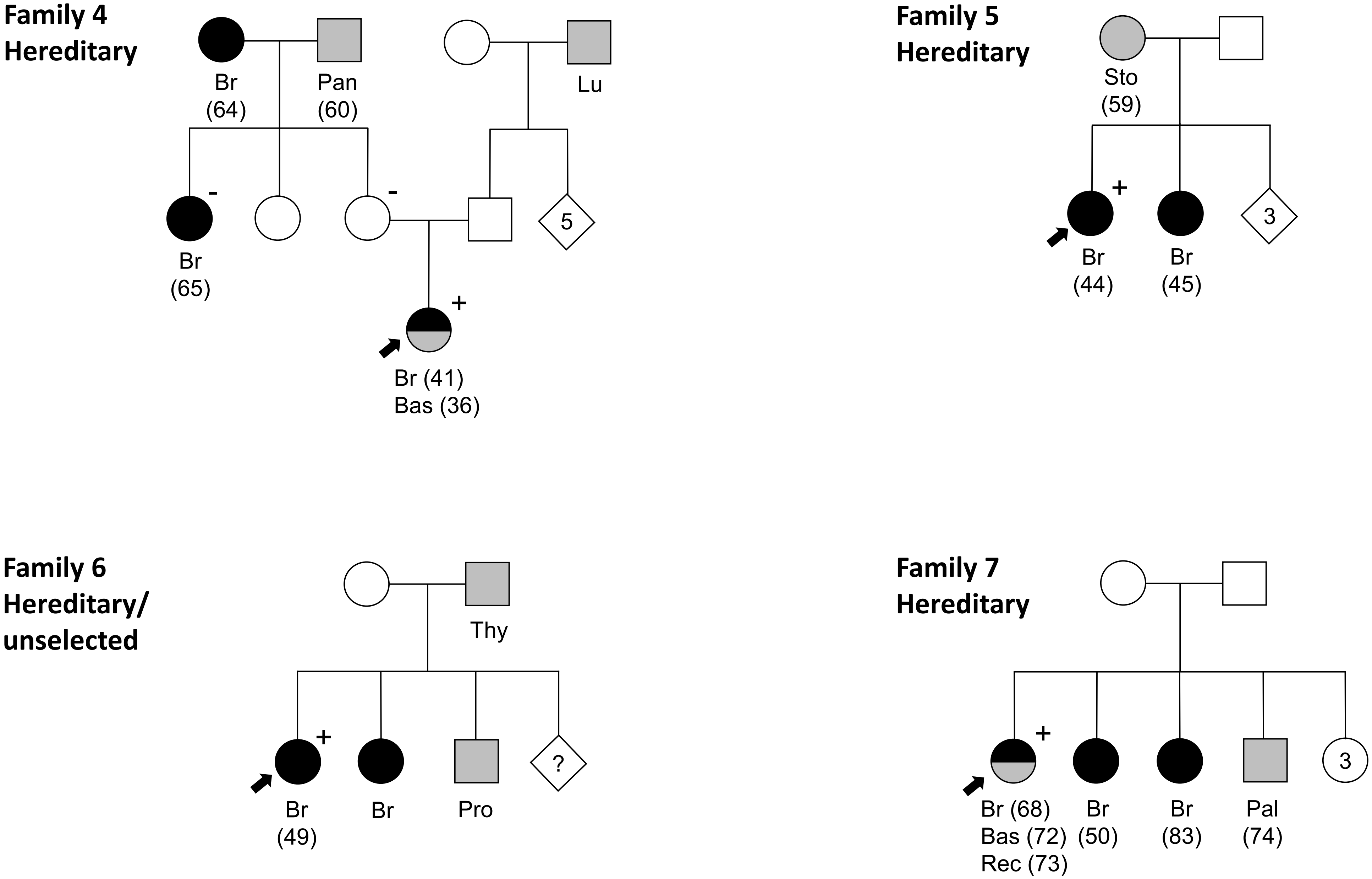

Supplementary figure S1. Pedigrees of **a)** the Northern Finnish *TEX15* c.7253dupT carriers and **b)** the Northern Finnish *FANCD2* c.2715+1G>A carriers and **c)** *FANCD2* c.2715+1G>A carriers from Helsinki. Next to each pedigree is the family ID and the cohort to which the index patient belongs. Breast cancer patients are marked with black and individuals with other cancers are marked with grey. Initially studied index patients are indicated with an arrow. Individuals genotyped for the studied mutations are marked with plus (carrier) or minus (non-carrier). The age at diagnosis, at the time of death or at last monitoring is below the individual if known, and deceased individuals are marked with a slash. Abbreviations: B1: individual genotyped for *BRCA1* c.4097-2A>G (rs80358019), Bas: basal cell carcinoma, Blood: blood cancer, Bm: bone marrow cancer, Br: breast cancer, Brain: brain tumor, Hep: hepatocarcinoma, Leu: leukemia, Lu: lung cancer, Ost: osteosarcoma, Ovca: ovarian cancer, Pal: palatine tonsil cancer, Pan: pancreatic cancer, Pro: prostate cancer, Rec: rectal cancer, Skin: skin cancer, Sto: stomach cancer, Thy: thyroid cancer, Tong: tongue cancer, Unk: unknown cancer cite, Ut: uterus cancer.

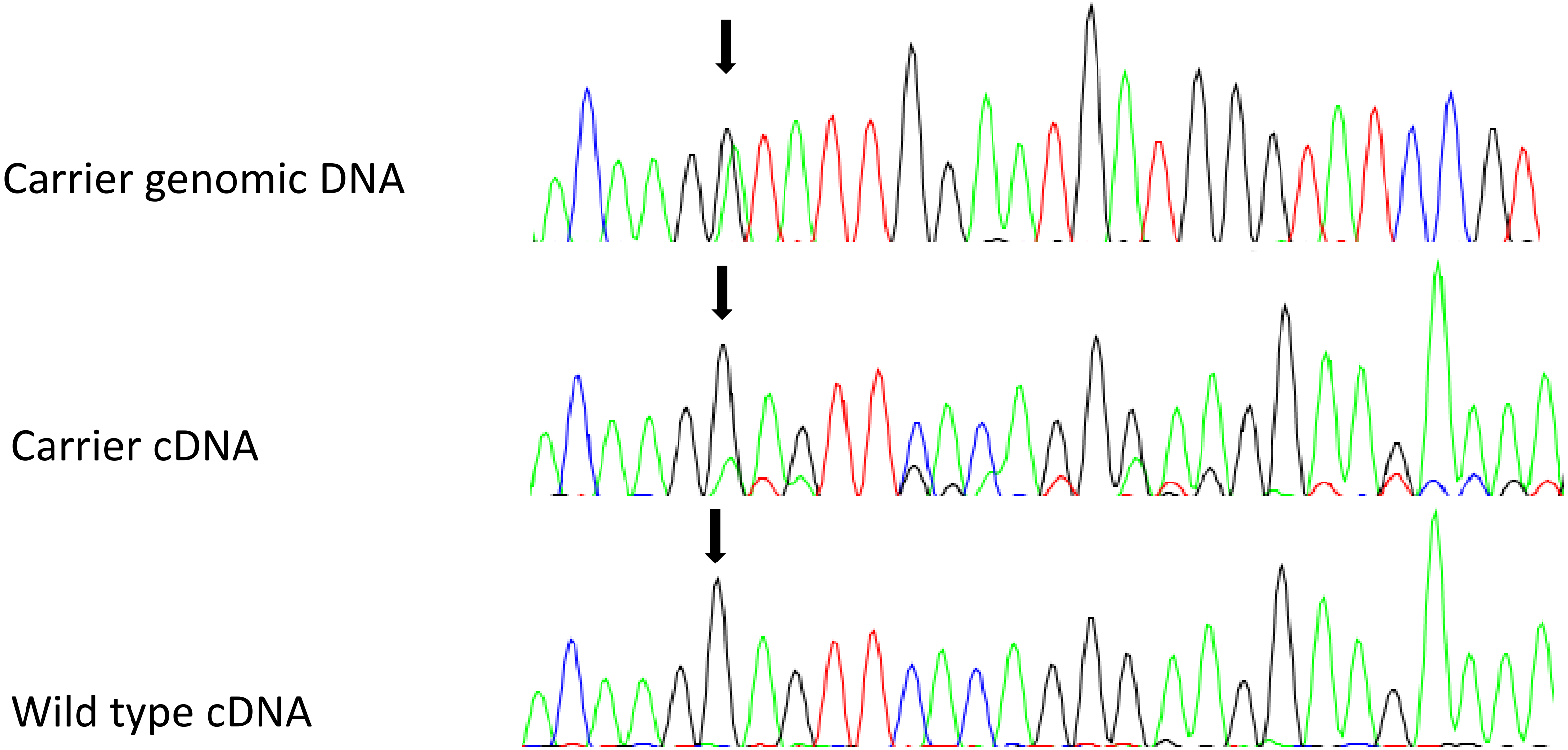

Supplementary figure S2. Sequencing of *FANCD2* c.2715+1G>A mutation site at cDNA and genomic DNA level.
